# Long Non-Coding RNAs in Pathogenesis of Renal Cell Carcinoma: Epigenetic Regulation, Signaling Pathways, and Therapeutic Strategies

**DOI:** 10.3390/ijms27115071

**Published:** 2026-06-03

**Authors:** Olga Savelieva, Irina Gilyazova, Anna Chumakova, Elza Khusnutdinova, Valentin Pavlov

**Affiliations:** 1 Laboratory of Molecular Genetics, Institute of Urology and Clinical Oncology, Bashkir State Medical University, 450008 Ufa, Russia; 2 Institute of Biochemistry and Genetics—Subdivision of the Ufa Federal Research Centre of the Russian Academy of Sciences (IBG UFRC RAS), 450054 Ufa, Russia

**Keywords:** renal cell carcinoma, long non-coding RNA, epigenetics, signaling pathways

## Abstract

Renal cell carcinoma (RCC) remains a major challenge in modern oncological urology, owing to its high heterogeneity, latent clinical course, and intrinsic resistance to chemotherapy and radiotherapy. In recent decades, the paradigm of carcinogenesis research has shifted from a primary focus on protein-coding genes alone to a broader investigation of the non-coding part of the transcriptome. Within this framework, long non-coding RNAs (lncRNAs) have emerged as fundamental regulators of cellular homeostasis. Accumulating evidence indicates that lncRNAs are not merely ‘transcriptional noise’ but components of intricate regulatory networks governing epigenetic, transcriptional, and post-transcriptional processes. Here, we present a comprehensive systematic review of the current literature addressing the role of lncRNA in the molecular pathogenesis of RCC. We discuss the roles of these molecules in key oncogenic signaling pathways, including PI3K/AKT/mTOR, Wnt/β-catenin, and Notch, and their contributions to tumor metabolic plasticity. The paper summarizes data on the link between lncRNAs and novel forms of regulated cell death—ferroptosis, cuproptosis, and disulphidosis. Particular attention is paid to their role in mediating resistance to tyrosine kinase inhibitors and their potential utility as highly specific biomarkers. Collectively, this review provides an updated perspective on the contribution of lncRNAs to RCC pathogenesis and outlines strategic directions for future research to support the development of more precise approaches in personalized oncology.

## 1. Introduction

Renal cell carcinoma (RCC) is one of the most common urological cancers worldwide, with more than 400,000 new cases diagnosed annually. The predominant histological subtype is clear cell renal cell carcinoma (ccRCC), which accounts for approximately 75% of verified RCC cases. This phenotype is characterized by aggressive clinical behavior and marked resistance to conventional radiation and chemotherapy. Moreover, ccRCC exhibits variable responses to targeted therapies and immunotherapy. Radical surgery is the mainstay of treatment; however, postoperative recurrence rates remain substantial, ranging from 20% to 50% [1]. The molecular pathogenesis of RCC is driven by the synergistic activation of oncogenes, inactivation of tumor suppressors, and dysregulation of key signaling pathways. RNA plays a central role in the transmission of genetic information, modulating gene expression and biological activity during neoplastic transformation [2].

Long non-coding RNAs (lncRNAs) are molecules longer than 200 nucleotides, primarily transcribed by RNA polymerase II. These molecules play a key role in the complex processes of genomic regulation. They interact with proteins, DNA, and RNA, organize the genome and cellular architecture, and coordinate finely tuned gene expression regulation [3]. Due to their capacity to modulate gene expression, lncRNAs are involved in a wide range of biological processes, including cell differentiation and development, programmed cell death, immune responses, bone and drug metabolism, and human reproductive function [4]. Based on genomic localization, lncRNAs are commonly classified into five categories: antisense, bidirectional, intronic, enhancer-associated, and intergenic. Intergenic and enhancer-associated lncRNAs possess independent promoters and are transcribed separately from protein-coding genes. By contrast, bidirectional RNAs share promoters with protein-coding genes but are transcribed from the opposite strand, whereas intronic lncRNAs originate from intronic regions of genes, including those associated with disease [3]. Accumulating evidence indicates that lncRNAs play critical roles in carcinogenesis, regulating tumor cell proliferation, invasion, metastasis, and drug resistance. However, their contribution to the development and progression of RCC remains insufficiently systematized and warrants comprehensive evaluation. Notably, lncRNAs are involved in key regulatory mechanisms in RCC, including epigenetic regulation and chromatin remodeling, and may serve as both robust biomarkers and potential targets for targeted therapeutic strategies.

The aim of this review is to systematically analyze and synthesize current evidence on the role of lncRNAs in the pathogenesis and progression of RCC, and to evaluate their potential as diagnostic, prognostic, and therapeutic biomarkers.

A systematic literature search was conducted in the PubMed (https://pubmed.ncbi.nlm.nih.gov, accessed on 20 March 2026), Scopus, and Web of Science databases using a combination of the keywords “long noncoding RNA,” “lncRNA,” “renal cell carcinoma,” and “kidney cancer,” using restrictions on publication date (2020–2026) and publication type (original studies and review articles). The initial search identified over 600 relevant accessed on 20 March 2026 publications. Studies were subsequently selected through a multi-stage screening process involving assessment of abstracts and full texts, complemented by a snowballing approach to identify additional relevant sources. Priority was given to studies with high-quality experimental design, including those with in vitro and in vivo validation, adequate sample sizes, and clear clinical correlations. In addition to conventional database searches, specialized lncRNA databases, including DIANA-LncBase v3.0 (https://diana.e-ce.uth.gr/lncbasev3/home, accessed on 20 March 2026), LncRNA2Target (http://bio-computing.hrbmu.edu.cn/lncrna2target/, accessed on 20 March 2026), and NONCODE (http://www.noncode.org/, accessed on 20 March 2026), were used to verify the annotations and functional significance of the selected transcripts.

## 2. Molecular Mechanisms of lncRNAs

### 2.1. Key Mechanisms of lncRNA Function

lncRNAs comprise a functionally heterogeneous class of transcripts exerting regulatory effects through a limited number of universal molecular strategies. Among the most prominent are the “scaffold,” “decoy,” and “guide” functions, which, in various combinations, enable lncRNAs to participate in the regulation of gene expression at multiple levels. As molecular scaffolds, RNAs facilitate the assembly of multicomponent ribonucleoprotein complexes, coordinating the spatial and functional interaction of enzymatic modules. The “decoy” function is based on the competitive binding of regulatory molecules, including miRNAs and proteins, which alter their availability and activity. The “guide” function of lncRNAs ensures specific targeting of protein complexes to specific genomic loci or RNA targets. Overall, lncRNAs act as integrators of molecular interactions, linking various levels of gene expression regulation—from epigenetic control to post-transcriptional processes [5].

### 2.2. Epigenetic Regulation and Chromatin Remodeling

**Figure 1:**
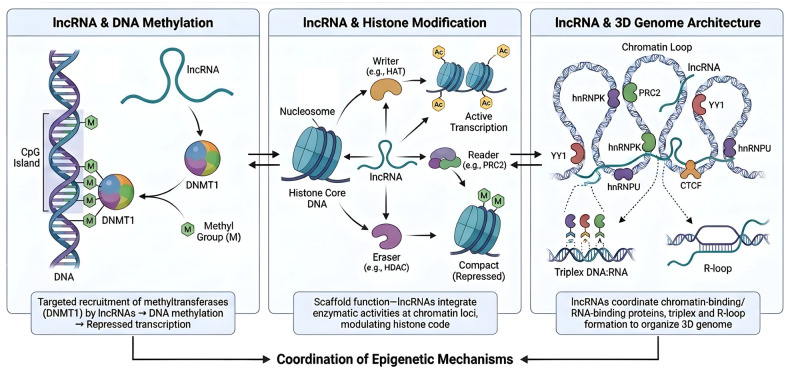
Multifaceted role of lncRNA in epigenetic regulation of gene expression.

Epigenetic regulation of gene expression encompasses mechanisms that modulate transcriptional activity without altering the DNA nucleotide sequence. lncRNAs are key regulators of epigenetic processes, coordinating DNA methylation, histone modifications, and chromatin organization. The functional role of lncRNAs involves the targeted recruitment of epigenetic factors and integration of various levels of regulation into a unified system to control transcriptional activity [6,7,8,9] (Figure 1).

DNA methylation is one of the primary mechanisms of epigenetic repression of transcription. lncRNAs are capable of directing DNA methyltransferases to specific genomic loci, thereby promoting the formation of methylated regions and maintenance of repressive chromatin. Experimental evidence indicates that interactions between lncRNAs and DNMT1 contribute to abnormal methylation of target genes [6].

In addition, lncRNAs play a central role in the regulation of histone modifications by interacting with enzymatic complexes—the ‘writers’, ‘erasers’, and ‘readers’ of the histone code. lncRNAs facilitate the targeted recruitment of these complexes to specific genomic loci, contributing to the formation of active or repressive chromatin states. Acting as molecular scaffolds, lncRNA molecules integrate various enzymatic activities, ensuring coordinated chromatin modification and the adaptation of the transcriptional program to the cellular context [7,8]. RNAs are also actively involved in the formation of the three-dimensional architecture of the genome, ensuring the spatial organization of chromatin. At this level of regulation, lncRNA interacts with chromatin-binding proteins, including hnRNPK, PRC2, YY1, and CTCF, as well as with RNA-binding proteins such as hnRNPU. Interaction mechanisms include mediated binding via protein complexes, the formation of triplex structures, Hoogsteen base pairing, and participation in the formation of R-loops —three-stranded RNA/DNA structures associated with transcriptional activity [9]. This multi-level organization provides coordination between various epigenetic mechanisms and the formation of stable chromatin domains.

Given the reversible nature of epigenetic modifications, chromatin-targeting therapeutic agents (epi-drugs) represent a promising frontier in modulating aberrant lncRNA expression and halting RCC progression. Small-molecule inhibitors—particularly histone deacetylase inhibitors (HDACis, such as vorinostat and panobinostat) and histone methyltransferase inhibitors—achieve therapeutic efficacy by correcting disordered chromatin states. In the context of RCC, highly progressive and metastatic phenotypes are frequently driven by oncogenic lncRNAs, such as *HOTAIR*, which interact with chromatin-modifying complexes like PRC2 and LSD1 to induce repressive histone modifications (e.g., H3K27me3) and silence tumor-suppressor genes. The application of specific EZH2 inhibitors (such as tazemetostat) or HDACis can disrupt these aberrant lncRNA–protein interactions, effectively prompting a global transcriptional reset. By preventing lncRNA-mediated remodeling of promoters and enhancers, these chromatin therapeutics can reactivate silenced tumor-suppressive transcripts, collapse oncogenic networks, and successfully impair RCC cell proliferation, angiogenesis, and metastatic colonization [7].

### 2.3. Transcriptional Regulation

**Figure 2:**
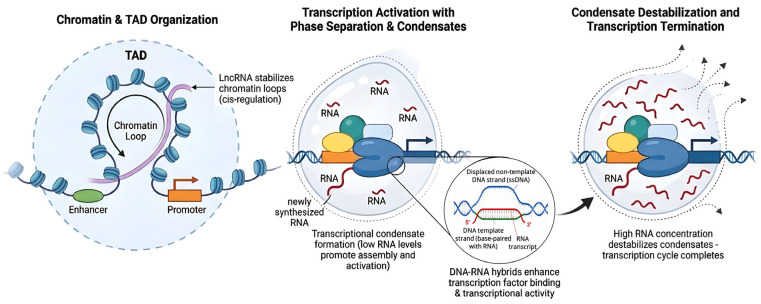
Model for transcriptional regulation: chromatin architecture and lncRNAs enable enhancer-promoter interactions within TADs, facilitating transcription activation.

Transcription is a complex enzymatic process that involves the synthesis of an RNA molecule using a DNA template and constitutes the primary stage of genetic information expression. DNA-RNA complexes act as fundamental integrators of regulatory signals, linking the three-dimensional architecture of chromatin to the functional activity of transcription mechanisms. In this capacity, they facilitate the physical proximity of distal enhancers to their target promoters within topologically associating domains, while also modulating the dynamics of RNA polymerase complexes to enable precise spatiotemporal control of gene expression. Genes with complex expression patterns are controlled by distal regulatory elements, such as enhancers, functioning within topologically associated domains. lncRNAs can stabilize chromatin loops between enhancers and promoters, thereby supporting cis-regulatory interactions and ensuring coordinated gene expression [6,10,11]. lncRNAs are also involved in the formation of transcriptional condensates via phase separation. A related mechanism is the RNA-mediated feedback model, where low transcript concentrations at early stages promote condensate assembly and activate transcription. Conversely, rising RNA concentration during elongation destabilizes these structures, triggering transcriptional termination [12]. The interaction of DNA-RNA hybrids with transcription factors enhances their binding to chromatin and increases transcriptional activity, despite limited specificity for RNA sequences [13] (Figure 2).

The functional integration of lncRNAs in RCC progression is tightly driven by modifications in their upstream transcriptional regulation. Under normal physiological states, basal transcriptional activation ensures well-balanced lncRNA expression. However, during pathological processes like oncogenesis, the transcriptional landscape undergoes severe alterations, leading to the abnormal and aberrant production of oncogenic lncRNAs. This transcriptional misregulation often involves the switch to alternative transcription start sites, differential isoform expression, and altered splicing patterns triggered by oncogenic stimuli and signaling pathways within the tumor microenvironment. In RCC, such disrupted mechanisms lead to the severe misexpression of key oncogenic transcripts—exemplified by progression-linked lncRNAs such as *LINC02532*. This widespread transcriptional chaos ultimately dismantles normal regulatory pathways and shifts cellular dynamics toward uncontrolled proliferation and advanced tumor progression [3].

### 2.4. Post-Transcriptional Regulation

Post-transcriptional regulation refers to the set of molecular mechanisms that control gene expression at the level of synthesized RNA molecules prior to their translation into proteins. Molecular studies confirmed that lncRNAs participate in post-transcriptional control by regulating alternative splicing, stability, transport and degradation of RNA [6,14,15,16,17,18,19,20,21,22].

Alternative splicing is a fundamental mechanism of spatial and temporal regulation of gene expression, ensuring exceptional proteomic diversity. The vast majority of genes (~95%) in human cells undergo alternative splicing. This process is tightly regulated by numerous RNA-binding proteins and splicing factors, including serine-/arginine-rich proteins and members of the heterogeneous nuclear ribonucleoprotein (hnRNP) family, as well as other factors containing specialized RNA-binding domains [6]. The main mechanisms used by lncRNAs to modulate alternative splicing may be divided into three groups: direct interaction of dsRNA with specific splicing factors, altering their availability or functional activity; the formation of complementary RNA-RNA duplexes with pre-mRNA molecules, which allow splicing sites to be physically masked. Furthermore, lncRNAs are capable of regulating splicing by altering transcription rates and chromatin state [14].

Most of the lncRNAs function as part of ribonucleoprotein complexes. RNA-binding proteins determine the fate and functions of lncRNAs by regulating their stability, intracellular transport and transcriptional activity. A key role in these processes is played by N6-methyladenosine (m6A) modification—the most common post-transcriptional modification of RNA. m6A influences the stability, transport and interactions of RNA with proteins. lncRNAs containing m6A modifications are involved in the regulation of oncogenic and tumor-suppressor pathways, acting as molecular ‘switches’ for cellular states [15,16,17,18].

The concept of competitive endogenous RNAs (ceRNAs), proposed by Salmena L. and colleagues, suggests the existence of regulatory networks whereby various transcripts, including lncRNAs, compete for binding to miRNAs via shared miRNA response elements (MREs) [19]. miRNAs are small (usually 19–25 nucleotides) single-stranded RNA molecules that do not encode proteins but play an important role in the regulation of gene expression. miRNAs are formed as a result of multi-step processing of primary transcripts (pri-miRNAs) involving the enzymes Drosha and Dicer and function as part of the RISC (RNA-induced silencing complex). miRNAs bind to MREs in the 3′-untranslated regions (UTRs) of target mRNAs primarily via complementarity of the ‘seed’ sequence (nucleotides 2–8 of the miRNA), leading to the suppression of gene expression through mRNA degradation or inhibition of protein translation [20]. According to the concept of competitive endogenous RNAs, lncRNAs function as molecular sponges, reducing the availability of miRNAs to their mRNA targets and thereby indirectly regulating gene expression [21]. Functionally, these lncRNA sponges often act as endogenous target mimics by exhibiting altered complementary sequences to specific miRNAs. Due to target-site mismatches that form structural bulges, these sponges recruit and sequester miRNAs without undergoing Ago2-mediated cleavage or degradation. This competitive entrapment shifts the homeostatic threshold of the regulatory network, successfully preventing the suppression of downstream target mRNAs [6]. The functioning of ceRNA networks is determined by the relative concentrations of miRNAs and competing transcripts, as well as the number and affinity of MREs. Once the system is sufficiently saturated, characteristic correlation relationships emerge: a negative correlation between miRNAs and their targets, and a positive correlation between transcripts competing for the same miRNA. Current data indicate that ceRNA interactions form complex, scalable, and dynamic networks, involving both direct and indirect effects resulting from competition for a shared pool of miRNAs. Disruptions to these networks play an important role in the pathogenesis of diseases, including oncogenesis, where dysregulation of ceRNA interactions leads to systemic changes in gene expression [21].

In addition to their regulatory functions, non-coding RNAs act as sources of small RNAs. A classic example is the *H19* non-coding RNA, which serves as a precursor for miR-675. This miRNA regulates the expression of the insulin-like growth factor receptor *Igf1r*, thereby influencing cell proliferation [22]. Such a mechanism represents a complex regulatory layer where lncRNAs facilitate the formation of small RNA cascades, significantly expanding the functional diversity of genomic regulatory networks [6].

## 3. Key Signaling Pathways Regulated by lncRNAs

### 3.1. PI3K/AKT/mTOR

The PI3K/AKT/mTOR signaling pathway acts as one of the central regulators of oncogenesis in RCC, acting as a critical hub integrating extracellular growth signals with processes of cell survival, metabolic reprogramming, and angiogenesis. Activation of this cascade drives extensive remodeling of the cellular phenotype [23,24,25,26,27,28,29,30]. Furthermore, this cascade engages in extensive crosstalk with other fundamental pathways, such as the Wnt/β-catenin and Notch axes, forming an integrated oncogenic network that synergistically accelerates tumor dissemination and drives therapy resistance [31,32,33,34,35,36,37,38,39,40,41,42,43,44,45,46,47].

One aspect of the functional activity of lncRNAs in RCC is their ability to directly modulate fundamental signaling pathways responsible for cell survival and metabolic reprogramming. A number of *LINC* family lncRNAs directly influence the phosphorylation of key kinases in the PI3K/Akt pathway. Evidence suggests that the expression level of *LINC00944* is significantly elevated in RCC cell lines (786-O, ASHN, OSRC-2, Ketr3) compared to the control. A unique feature of this molecule is that its knockdown using CRISPR/dCas9-KRAB in 786-O cells not only reduces cell proliferation and migration but also leads to a paradoxical increase in Akt phosphorylation, indicating a complex, likely context-dependent, regulatory role in this cascade [23]. In contrast, *LINC00460* functions as a classic oncogene. Knockdown of this RNA in ACHN and 786-O cells inhibits oncogenic potential and reduces the expression of key components of the PI3K/AKT/mTOR axis [24] (Table 1).

lncRNAs regulate key points of the cell cycle and protein ubiquitination, processes that are critically involved in the modulation of the PI3K/AKT/mTOR signaling pathway. A specific role as an oncogene was identified for *RCAT1* (Renal cancer-associated transcript 1), the expression of which is significantly elevated in RCC tissues and cells. Mechanistically, this effect is mediated via the *RCAT1*/miR-214-5p/*E2F2* regulatory axis. *RCAT1*, acting as a molecular sponge, blocks the activity of miR-214-5p, leading to the stabilization and accumulation of the transcription factor E2F2. Elevated levels of E2F2 promote the transition of cells to an accelerated proliferation cycle and simultaneously suppress apoptosis by reducing the expression of pro-apoptotic factors such as p53 and BAX, which, in combination with background activation of the PI3K/AKT pathway, ensures the survival and aggressive metastasis of tumor cells [25]. Similarly, *FTX* promotes RCC progression via the *FTX*/miR-4429/*UBE2C* axis. *FTX* overexpression in cell lines leads to increased expression of the ubiquitin-conjugating enzyme UBE2C. It is accompanied by the activation of the cyclin-dependent kinases CDK1 and CDK6, as well as the PI3K/AKT/mTOR signaling pathway. Consistent with these findings, tumors characterized by *FTX* overexpression exhibit significantly increased mass and volume [26].

Regulatory RNAs capable of altering the tumor’s immune landscape play a key role in modulating the activity of the classical PI3K/AKT/mTOR signaling pathway in RCC. Xu P. et al. found that the *AGAP2-AS1* plays a key role in the development of a pro-tumor microenvironment in ccRCC by stimulating M2 polarization of macrophages. Mechanistically, *AGAP2-AS1* functions as a miRNA sponge for miR-9-5p, increasing the expression of THBS2 protein and activating the PI3K/Akt signaling pathway. A series of in vitro experiments on cell lines (786-O, Caki-1) showed that the RNA-binding protein IGF2BP3 stabilizes *AGAP2-AS1* by recognizing m6A modifications, thereby preventing its degradation. In vivo experiments confirmed that in mice injected with ACHN cells lacking *AGAP2-AS1*, tumor volume and size were significantly reduced [15].

In a number of cases, lncRNAs promote integration of the PI3K/AKT/mTOR pathway with other oncogenic cascades, forming stable regulatory networks that sustain the uncontrolled proliferation of RCC cells. Yang Y. et al. reported that elevated expression of *FGD5-AS1* in RCC tissues is associated with distant metastasis and an aggressive tumor phenotype. Analysis of RCC cell lines showed that *FGD5-AS1* functions as a molecular sponge for miR-5590-3p, resulting in activation of the ERK/AKT signaling pathways, which are critical regulators of the cell cycle and survival. Since the AKT kinase is a central hub of the PI3K/AKT/mTOR cascade, activation of this axis initiates extensive metabolic reprogramming and releases the mTORC1 complex, which is responsible for protein biosynthesis and cell survival [27]. Kulkarni P. et al. identified that *TCL6* acts as a tumor growth suppressor in ccRCC, regulating metastatic potential via the miR-155/Src/Akt axis. A deficiency in *TCL6* was found to lead to the overexpression of miR-155, causing the activation of the Src/Akt signaling pathway, which is closely linked to the PI3K/AKT/mTOR pathway, and the initiation of EMT in the 786-O, A498, and HK-2 proximal tubule epithelial cells. Ectopic restoration of *TCL6* in RCC cells was observed to induce apoptosis and inhibit migration, confirming the functional role of this molecule in the suppression of tumor growth [28]. Xie G. et al. revealed that the expression level of *PVT1,* which promotes the proliferation of RCC cells (Caki-1), is significantly higher in RCC tissues compared to controls. *PVT1* sequesters miR-328-3p via a competing endogenous RNA mechanism, leading to increased expression of the *FAM193B* gene and activation of the MAPK/ERK and PI3K/AKT signaling pathways [29]. Tan et al. found that the *GABPB1-IT1* acts as a sponge for miR-21, preventing its inhibitory effect on the tumor suppressor *PTEN*. *PTEN* is the main negative regulator of the PI3K/AKT/mTOR pathway. Restoration of *PTEN* expression via this mechanism leads to the suppression of Caki-2 cell proliferation, suggesting the potential for pharmacological modulation of the *GABPB1-IT1*/miR-21/*PTEN* axis to inhibit tumor growth [30].

### 3.2. Wnt/β-Catenin

The Wnt/β-catenin signaling pathway has a central role in the regulation of cell proliferation, differentiation, and adhesion, and is widely recognized as a key driver of tumor progression in RCC. In the canonical pathway, activation of Wnt ligands leads to the stabilization of β-catenin, its accumulation in the cytoplasm, and subsequent translocation to the nucleus, where it interacts with transcription factors of the TCF/LEF family and induces the expression of genes involved in cell growth and survival. Dysregulation of the Wnt pathway contributes to enhanced proliferation, invasion, and EMT in RCC. The regulatory influence of lncRNA on the Wnt/β-catenin cascade is exerted at various levels, ranging from the modulation of the availability of extracellular ligands to the stabilization of membrane receptor complexes [31,32,33,34,35,36,37,38].

Hu Z. et al. identified significantly higher levels of *MSC-AS1* expression in ccRCC tissues compared to controls, correlating with an unfavorable prognosis. The results indicate that *MSC-AS1* functions as a ceRNA, binding to miR-3924 and increasing the expression of the WNT5A ligand. *MSC-AS1* silencing effectively suppressed the proliferation and migration of 786-O and 769-P RCC cells. Consistently, in vivo xenograft models revealed a significant reduction in tumor volume following *MSC-AS1* knockdown compared to control groups. Overall, activation of the *MSC-AS1*/miR-3924/WNT5A axis leads to sustained stimulation of the Wnt/β-catenin pathway, which enhances the proliferation and migratory activity of RCC cells [32]. In vitro (769-P and Caki-1) and in vivo experiments showed that knockdown of the *NLGN1-AS1* inhibits cell proliferation and RCC growth. Luciferase reporter assays proved that *NLGN1-AS1* acts as a ceRNA by binding to miR-136-5p and preventing lncRNA-mediated degradation of the FZD4 receptor. Activation of the *NLGN1-AS1*/miR-136-5p/FZD4 axis leads to the stabilization of β-catenin and the initiation of a Wnt-dependent transcriptional program [33]. Mao et al. found that *SLERCC* expression was significantly reduced in human RCC tissue samples compared with normal tissue. *SLERCC* functions as a tumor suppressor via the Wnt/β-catenin pathway [34].

In addition to highly specific effects, several lncRNAs demonstrate the ability to exert integrative regulation, simultaneously modulating the Wnt/β-catenin cascade and associated oncogenic pathways. Cheng G. et al. detected a significantly higher expression of *LINC00160* lncRNA in RCC, correlating with a poor prognosis. Knockdown of *LINC00160* leads to suppression of the malignant phenotype of cells in vitro (ACHN, 786-O) and inhibition of tumor growth in vivo. *LINC00160* exhibits a distinct capacity to epigenetically regulate genomic loci in both cis and trans. It is implicated in the modulation of the Wnt and mTOR signaling pathways, the cell cycle, and fatty acid metabolism; however, the precise molecular mechanisms coordinating these cascades remain to be elucidated [35]. Functional studies found that ectopic overexpression of *LINC01939* inhibits the proliferation and migration of RCC cells (ACHN and Caki-1), as well as inducing their apoptosis. The molecular mechanism is based on the sequestration of miR-154 by *LINC01939*, leading to synergistic inactivation of the Wnt/β-catenin and Notch signaling pathways. The involvement of *LINC01939* as a key regulator of several oncogenic cascades indicates its significant tumor-suppressive potential [36]. Yu H. et al. identified overexpression of *SNHG12* in ccRCC tissues, directly correlating with an unfavorable clinical outcome. In vitro functional assays in RCC cell lines (786-O and OS-RC-2) revealed that *SNHG12* knockdown significantly inhibits cell proliferation and migration. *SNHG12* acts as a competing endogenous RNA for miR-30a-3p, leading to the activation of a whole cascade of target genes—*RUNX2*, *WNT2,* and *IGF-1R*—that promote tumor progression [37].

The integration of bioinformatics data with functional assays allowed for the identification of a number of lncRNAs possessing pronounced anti-oncogenic properties. Zhu et al. found that the expression level of *LINC00675* was significantly reduced in RCC tissues and cell lines. Experimental data validated the anti-oncogenic properties of *LINC00675*, demonstrating that its ectopic expression reduces the proliferative, migratory, and invasive capacity of 786-O and 769-P cells. The study revealed that in both cell lines, against a background of increased *LINC00675* expression, there is a reduction in the level of β-catenin, which is a key component of the Wnt/β-catenin signaling pathway. Although previous studies suggest that *LINC00675* acts as a ceRNA for miR-942, the precise mechanism by which this RNA exerts its effects in RCC via modulation of Wnt signaling requires further clarification [38].

### 3.3. Notch

The Notch signaling pathway is a key regulator of cell differentiation, proliferation, and survival. Dysregulation of this pathway is closely linked to oncogenesis and the development of drug resistance. The key components of the Notch signaling pathway are four receptors (Notch1, Notch2, Notch3, and Notch4), five ligands (JAG1, JAG2, DLL1, DLL3, and DLL4), and several target genes. Receptor-ligand binding stimulates activation of the Notch signaling pathway, followed by hydrolysis of Notch receptors by *ADAM* and γ-secretase, resulting in the formation of the Notch intracellular domain (NICD), involved in the regulation of transcription of downstream genes such as *HES1*, *HEYL*, and *KAT2A* [39].

An important mechanism for epigenetic regulation of the Notch cascade is the direct interaction of lncRNAs with chromatin components. Wang Y. et al. found reduced expression of the *MIR503HG* in papillary renal cell carcinoma (pRCC) tissues, caused by hypermethylation of the gene’s promoter region. On the other hand, *MIR503HG* binding to the histone variant H2A.Z modulates its recruitment to chromatin, inducing H3K27 trimethylation (H3K27me3). Enhancement of the repressive H3K27 mark suppresses *NOTCH1* gene transcription, leading to reduced secretion of the VEGFC factor and inhibition of lymphangiogenesis. Conversely, *MIR503HG* deficiency promotes the expression of the HNRNPC protein, stimulating the processing of *NOTCH1* mRNA, thereby accelerating tumor progression [40].

lncRNAs are critical regulatory hubs ensuring aberrant persistence of Notch signaling and counteracting the anti-proliferative effects of targeted therapy and chemotherapy. Liu S. et al. demonstrated that the *RP11-567G11.1* activates the malignant progression of RCC. Knockdown of *RP11-567G11.1* significantly reduces the proliferative and invasive activity of RCC cells (786-O and 769-P) and also suppresses the expression of key effectors of the Notch signaling pathway, Jagged1, HES5, and HEY1. The findings suggest that functional inhibition of *RP11-567G11.1* induces apoptosis and enhances the sensitivity of cells to cisplatin, defining it as a potential target to overcome chemoresistance [41].

The prognostic potential of aberrant Notch signaling is also reflected in the development of multi-gene signatures. Profiling of associated lncRNAs serves as a reliable tool for accurate patient stratification and the prediction of clinical outcomes in ccRCC. Zhang L. et al. established that a specific expression pattern of five lncRNAs functionally associated with Notch signaling (*AC092611.2*, *NNT-AS1*, *AGAP2-AS1*, *AC147651.3* and *AC007406.3*), is an independent prognostic biomarker for ccRCC. The developed prognostic signature allows for effective stratification of patients into risk groups. Functional analysis revealed that this model possesses high predictive power not only for overall survival but also for tumor response to targeted and immunotherapy [39].

### 3.4. Other Signaling Pathways

The intracellular signaling network in RCC extends beyond the pathways described above to encompass several additional cascades that modulate disease progression. The JAK3/STAT3 signaling pathway is an important oncogenic cascade responsible for tumor cell survival, proliferation, and the formation of an immunosuppressive microenvironment. Zhang W.C. et al. showed that activation of the canonical JAK/STAT pathway by exosomal lncRNA is a critical factor in the epigenetic reprogramming of macrophages, contributing to their transformation into a pro-tumor state. Evidence suggests that RCC cells remotely modulate macrophage phenotypes via the exosomal transfer of *ARSR*, which acts as a ceRNA by sequestering miR-34 and miR-449 [42].

On the other hand, the development of RCC is largely associated with the depletion of effector lymphocytes and the conversion of anti-tumor immunity into a state of functional inactivity. Studies of humanized PDX models showed that high levels of *MIAT* expression correlate directly with the functional exhaustion of tumor-infiltrating CD8+ T-lymphocytes. The molecular mechanism underlying this effect is associated with epigenetic modulation of JAK/STAT signaling. Specifically, nuclear *MIAT* complexes with the ETS1 transcription factor, thereby stimulating *JAK3* kinase expression and subsequent activation of the entire JAK3/STAT3 pathway. The data indicate that suppression of *MIAT* in RCC cells (769-P and 786-O) restores the effector functions of T cells and reduces the expression of exhaustion markers PD-1 and TIGIT [43]. *LINC01605* mediates immune evasion by modulating membrane glycosylation, a process that drives tumor progression and leads to the depletion of the CD8+ T-cell pool. At the molecular level, *LINC01605* interacts with the IGF2BP2 transporter protein, increasing the stability of JAK3 kinase mRNA. High expression of JAK3 activates the JAK3/STAT3 signaling pathway, whereby phosphorylated STAT3 induces the transcription not only of classical oncogenes (e.g., *MYC*), but also the sialyltransferase ST6GALNAC5. This enzyme directly increases the sialylation of cell membranes, creating a glycosylation-based ‘shield’ that prevents tumor recognition by immune cells. This phenomenon is supported by the fact that *LINC01605* knockdown in RCC cells (A498 and 786-O) significantly reduces the total level of sialic acid in membranes, restoring the immunogenicity of the cells [44].

The integration of metabolic reprogramming with systemic regulation of cellular plasticity is mediated by RNAs that serve as central hubs for the cross-regulation of signaling pathways. Yin W. found that the purine metabolism-associated RNA *LINC01671* suppresses the progression of ccRCC. Ectopic overexpression of *LINC01671* was reported to inhibit the proliferation and migration of Caki-1 cells while promoting apoptosis. Gene Set Enrichment Analysis (GSEA) revealed that its anti-tumorigenic effects are mediated through the multimodal modulation of key signaling cascades, including the MAPK, NF-κB, mTOR, PI3K-Akt, and Wnt pathways [45]. Conversely, elevated levels of *LUCAT1* in RCC tissues and cells promote the sequestration of miR-375, thereby increasing *YAP1* expression and activating the Hippo pathway. In vitro experiments (Caki-1, A498) showed that knockout of *LUCAT1* suppresses cell proliferation and invasion, whereas inhibition of miR-375 or overexpression of YAP1 restores the malignant phenotype. In vivo experiments validated that *LUCAT1* suppression effectively inhibits xenograft growth. In general, the *LUCAT1/*miR-375/*YAP1* axis destabilizes the contact-inhibited division system characteristic of normal renal epithelium [46].

*LINC02609* contributes to the regulation of canonical proliferative cascades, with its expression in tumor tissue correlating positively with disease aggressiveness. Xing C. et al. established that *LINC02609* expression is significantly elevated in RCC tissues and is associated with a negative prognosis. In vitro experiments using A498 cells showed that suppression of *LINC02609* inhibits proliferation, migration, and invasive potential. Mechanistically, these effects are mediated via positive regulation of the mitogen-activated protein kinase (MAPK) signaling pathway, a central regulator of cell-cycle progression and survival in RCC; knockdown of *LINC02609* reduced phosphorylation of key effectors such as ERK1/2, supporting its role as a promoter of MAPK-dependent cancerogenesis. In particular, *LINC02609* knockdown leads to reduced phosphorylation levels of key cascade effectors (ERK1/2), confirming the role of this lncRNA as a primary activator of MAPK-dependent oncogenesis [47].

## 4. Regulation of Cell Cycle Progression and Mechanisms of Programmed Cell Death

### 4.1. Cyclin and CDK-Dependent Checkpoints

The regulation of the cell transition to active division and the maintenance of the cancer stem cell population are fundamental processes controlled by specific RNAs. The *NORAD*, which stabilizes expression of MYC-family oncogenes, has been implicated in modulating RCC aggressiveness. Experiments on RCC cell lines (ACHN and A498) showed that *NORAD* stimulates cell proliferation and migration by acting as a selective sponge for miR-144-3p, preventing the suppression of its target, the *MYCN* oncogene. It belongs to the *MYC* family of oncogenes, acting as cell cycle regulators that directly control the expression of cyclins and CDKs. The validity of the *NORAD*/miR-144-3p/*MYCN* axis functions was proven in rescue experiments, where co-expression of miR-144-3p partially counteracted the malignant phenotypic transformation caused by excess *NORAD* [48] (Table S1).

RCC pathogenesis is predominantly driven by lncRNAs that coordinate survival and dedifferentiation programs through direct influence on the cell division mechanism. One example is *EMBP1,* which functions as an important regulator of carcinogenesis and suppresses the activity of the tumor-suppressor miR-9-5p. Experiments on RCC lines (786-O and Caki-1) showed that *EMBP1* suppression or miR-9-5p overexpression induces apoptosis and inhibits tumor growth. The *EMBP1*/miR-9-5p axis functions as a central regulator, coordinating multiple critical programs, including the expression of EMT markers (E-cadherin, claudin, vimentin), pluripotency factors (KLF4, Nanog), and the cell cycle checkpoint gene *CCNE2*/*E2F1*. In vivo experiments confirmed that the inhibitory effect of *EMBP1* suppression is completely negated by CCNE2 overexpression, demonstrating the key role of this pathway in enabling RCC cells to progress through the G1/S phase [49].

Direct regulation of D-type cyclin biogenesis is one of the most common mechanisms by which lncRNAs drive tumor cells into the synthetic phase. Experiments on RCC cell lines (786-O and Caki-1) showed that overexpression of the *TTN-AS1* stimulates proliferation and accelerates the G1/S transition of the cell cycle. *TTN-AS1* functions as a ceRNA by binding the tumor suppressor miR-195, thereby preventing the suppression of the miRNA’s direct target, cyclin D1. Rescue experiments have confirmed that increasing miR-159 levels partially blocks the oncogenic effects of *TTN-AS1*, demonstrating the critical importance of the *TTN-AS1*/miR-195/Cyclin D1 axis for aggressive tumor growth [50].

In addition to controlling the onset of the synthetic phase, lncRNAs play a crucial role in ensuring the accuracy and speed of the division process itself by regulating the assembly of the mitotic apparatus. These mechanisms are of particular significance in aggressive histological subtypes of the disease, in which impaired mitotic control correlates directly with survival. Ding Z. et al. found that the TPX2 protein and the *LINC00894* form a key oncogenic axis that drives the progression of pRCC, particularly its aggressive phenotype. Analysis of clinical pRCC samples and cell lines (Caki-2, ACHN) identified a strong correlation between high TPX2 expression and both poor prognosis and reduced survival rates. Mechanistically, *LINC00894* acts as a competitive endogenous RNA for miR-660-5p, thereby preventing the degradation of *TPX2* mRNA. Accumulation of the TPX2 protein, which is involved in spindle assembly, enhances the proliferation and migration of cancer cells [51].

Beyond classical ceRNA mechanisms, control over checkpoints can also be exerted through the modulation of transcription factors that coordinate the expression of an entire kinase clusters. Tang Z. et al. demonstrated in experiments on Caki-2 RCC cells that overexpression of *LINC00478* enhances invasion and metastasis by activating downstream cell cycle regulators such as CDCA8 and CDK2. *LINC00478* has been found to bind to the oncogene *PBX3*, promoting its transcription and translation. This interaction leads to the up-regulation of downstream cyclins, which critically accelerates tumor cell division and enhances invasive potential. These findings highlight the role of *LINC00478* as a key regulator of cell cycle progression and metastasis [52].

### 4.2. Apoptosis as a Response to Cell Cycle Disorders

**Figure 3:**
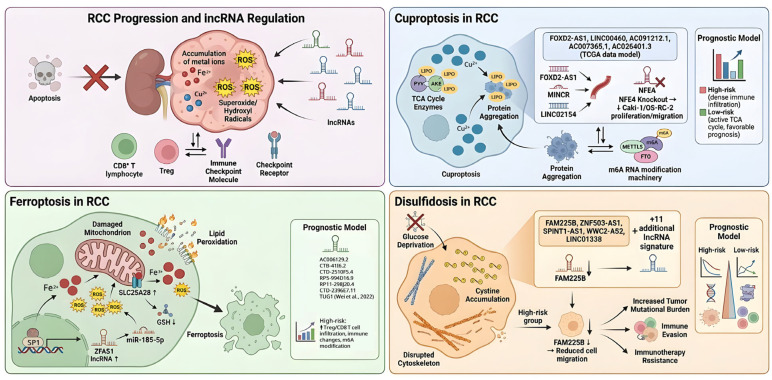
Mechanisms of programmed cell death and lncRNA-based prognostic models in RCC [16].

Apoptosis acts as a fundamental protective barrier, initiating a program of cell self-destruction in response to critical errors in DNA replication or defects in the assembly of the mitotic apparatus. Specific lncRNAs coordinate mechanisms to evade programmed cell death in the pathogenesis of RCC, enabling tumor cells to disregard signals of checkpoint disruption and maintain viability even under conditions of pronounced genomic instability (Figure 3).

Apoptosis in RCC is largely regulated by suppression of pro-apoptotic lncRNAs. The *PCGEM1* functions in a similar manner. The data provide evidence that knockdown of *PCGEM1* in vitro (786-O and A498 cells) leads to suppression of proliferation and enhanced apoptosis. *PCGEM1* competitively binds miR-433-3p, resulting in increased expression of the FGF2 growth factor and providing autocrine support for survival [53]. A similar pattern of survival dependence upon the level of non-coding transcripts was described for *LINC00641*. Zhang J. et al. showed that knockdown of *LINC00641* suppresses the proliferation, colony-forming ability, and invasion of RCC cells (A498, ACHN), simultaneously inducing their apoptosis. In vivo experiments in mouse models established that inhibition of *LINC00641* leads to a reduction in tumor volume through binding to miR-340-5p, acting as a tumor growth suppressor [54].

The family of small nuclear RNA host genes (*SNHG)*, playing a central role in anti-apoptotic networks, makes a significant contribution to the development of RCC cell resistance to apoptosis. Wu J. et al. found that *SNHG4* actively suppresses apoptosis by directly reducing the activity of effector caspases-3, -8, and -9, and promotes invasion. This biological effect is mediated by the sequestration of miR-204-5p, leading to the enhancement of the expression of the RUNX2 transcription factor, which is responsible for the aggressive behavior and resistance of RCC cells to death signals. In vivo studies on xenotransplant models in mice confirmed that *SNHG4* promotes tumor growth [55]. Xu Z. et al. showed that suppression of *SNHG3* inhibits tumor progression in RCC cell lines (A498 and 786-O). *SNHG3* functions as a sponge for miR-10b-5p, preventing the degradation of the anti-apoptotic factor BIRC5 [56]. Significantly higher levels of *SNHG3* expression were found in ccRCC tissues compared to normal tissue, according to TCGA (The Cancer Genome Atlas) data. Evidence suggests that *SNHG3* expression levels correlate with immune cell infiltration in ccRCC and sensitivity to various targeted and chemotherapeutic agents. The study revealed that *SNHG3* knockdown significantly reduces the proliferation and migration of RCC cells (786-O) [57].

The *SNHG12* plays a key role in the RCC pathogenesis by activating several independent oncogenic cascades simultaneously, depending on the tumor microenvironment. Xu C. et al. identified *SNHG12* overexpression in RCC tumor tissues, which directly correlates with a poor prognosis. Lentiviral-mediated *SNHG12* silencing in A498 and 786-O cell lines significantly reduced viability and invasiveness while simultaneously triggering apoptosis. *SNHG12* acts as a sponge for miR-200c-5p, resulting in increased expression of the collagen XI type α1 chain gene *COL11A1* [58]. Feng et al. found that the *SNHG12* acts as a critical promoter of RCC growth and angiogenesis, under the epigenetic control of the KMT2B methyltransferase. Specifically, KMT2B-mediated H3K4me3 modification elevates *SNHG12* levels, enabling the lncRNA to recruit E2F1 and subsequently activate *CEP55* oncogene transcription. The results indicate that blocking the KMT2B/*SNHG12*/E2F1/*CEP55* functional axis by sequential knockdown of individual components leads to a decrease in histone H3K4me3 methylation levels in the lncRNA promoter, which effectively suppresses the recruitment of the E2F1 transcription factor and the subsequent activation of the *CEP55* oncogene, thereby inhibiting RCC cell proliferation and neoangiogenesis [59].

The resistance of RCC cells to apoptotic stimuli is assured not only by the direct inhibition of the caspase cascade, but also by the systemic involvement of *SNHG* family lncRNAs in maintaining cellular structural integrity. These transcripts form a protective barrier by coordinating the stability of the mitotic apparatus and an adequate response to genotoxic stress, allowing tumor cells to avoid death even in the face of critical DNA damage. Cheng T. et al. identified significant overexpression of *SNHG16* in RCC patient tissues and cell lines (786-O, A498). *SNHG16* suppression effectively inhibits proliferation and induces apoptosis in A498 and 786-O cells. Mechanistically, *SNHG16* functions as a competing endogenous RNA for miR-1301-3p, thereby preventing the suppression of the *STARD9* gene, which drives RCC cell growth and survival [60]. A study by Wu J. et al. determined that *SNHG17* promotes tumor survival by modulating factors involved in the response to genotoxic stress. *SNHG17* overexpression in RCC tissues and cell lines (786-O and ACHN) was associated with more advanced disease stages, as well as reduced recurrence-free and overall survival. In vitro and in vivo functional experiments demonstrated that *SNHG17* knockdown suppresses proliferation, migration, and invasion, and induces apoptosis in RCC cells. In this case, *SNHG17* functions as a molecular sponge for miR-328-3p, stabilizing H2AX histone expression [61].

The ability of tumor cells to evade programmed cell death is closely linked not only to the regulation of cell division but also to the maintenance of the stability of the translational machinery. Under normal conditions, a deficiency in key components of protein synthesis acts as a potent pro-apoptotic stimulus; however, specific antisense transcripts are capable of counteracting this response. An important mechanism of this defense is the activation of antisense RNAs involved in tRNA synthetase function, including the antisense RNA 1 to aspartyl-tRNA synthetase *DARS-AS1* RNA. Functional assays showed that silencing of *DARS-AS1* in RCC cells (786-O and Caki-1) inhibits the cell cycle and induces apoptosis. The findings suggest that *DARS-AS1* is localized predominantly in the cytoplasm and functions as a sponge for miR-194-5p, preventing post-transcriptional repression of the target *DARS* oncogene. The *DARS* gene encodes a protein responsible for the synthesis of aspartyl-tRNA, necessary for maintaining a high rate of protein synthesis in a growing tumor [62]. Wang et al. found that the *CYTOR* acts as a potent oncogene in RCC, promoting cell escape from apoptosis and enhancing their invasive potential. Available data point to *CYTOR* regulates *MAT2B* expression through competitive binding of miR-136-5p, and the MAT2B protein, in turn, interacts with the anti-apoptotic factor BAG3. The data provide evidence that *CYTOR* knockdown effectively inhibits tumor growth in vivo, making the *CYTOR*/miR-136-5p/*MAT2B* axis a promising target for therapeutic intervention [63].

### 4.3. Ferroptosis, Curroptosis, and Disulfidosis

Current understanding of the RCC progression involves mechanisms to escape not only caspase-dependent cell death but also metabolic catastrophes induced by the accumulation of reactive oxygen species and metal toxicity.

Ferroptosis is a genetically and biochemically determined form of programmed cell death characterized by iron-dependent accumulation of reactive oxygen species and subsequent lipid peroxidation. In contrast to apoptosis, ferroptosis proceeds without caspase activation or DNA fragmentation, and its morphological features include a reduction in mitochondrial volume, an increase in mitochondrial membrane density, and a reduction in mitochondrial cristae. The systemic significance of ferroptosis in RCC is confirmed by the development of comprehensive biomarker models linking iron metabolism to the state of local immunity (Figure 3). Wei S.Y. et al. developed a prognostic model based on a signature of seven lncRNAs associated with ferroptosis, allowing for the assessment of ccRCC patient survival. High-risk patients were characterized by increased infiltration of regulatory T cells (Tregs) and CD8+ T lymphocytes, alongside significant alterations in immune checkpoint gene expression and m6A modification machinery. The study results confirm the existence of a link between ferroptosis mechanisms and the tumor immune microenvironment, proposing a new model for personalized prognosis of disease outcomes [16]. In addition to their prognostic role, certain lncRNAs act as direct mediators of the ferroptotic cascade in response to metabolic stress. Tao Q. et al. reported that *ZFAS1* lncRNA promotes ferroptosis in ccRCC through regulation of the *ZFAS1/*miR-185-5p/*SLC25A28* axis. In vitro and in vivo experiments showed that the transcription factor SP1 activates *ZFAS1* expression in response to metabolic stress, leading to the sequestration of miR-185-5p and a subsequent increase in SLC25A28 protein expression. Activation of this cascade causes the accumulation of ROS and depletion of the antioxidant pool, particularly glutathione (GSH), which significantly enhances tumor cell death [64].

In recent years, researchers’ attention has focused on cuproptosis, a copper-dependent form of programmed cell death associated with the lipoylation of proteins in the tricarboxylic acid cycle. LncRNAs act here not only as regulators but also as predictors of tumor sensitivity to copper ion toxicity. The clinical significance of this process in RCC is confirmed by the development of both multigene prognostic scales and the identification of individual markers of response to therapy (Figure 3). Zhang W. et al. developed a prognostic model based on five lncRNAs associated with cuproptosis (*FOXD2-AS1*, *LINC00460*, *AC091212.1*, *AC007365.1*, *AC026401.3*) based on an analysis of TCGA data. This model allows for the assessment of survival in clear ccRCC. Crucially, a high risk on this scale is associated with intense immune infiltration, whereas in low-risk patients, the tricarboxylic acid cycle remains active, and a more favorable prognosis is observed [65]. Katifelis H. et al. reported that the expression levels of the cuproptosis-associated lncRNAs *FOXD2-AS1*, *MINCR*, and *LINC02154* in peripheral blood are promising predictors of response to immunotherapy in metastatic ccRCC. Specifically, elevated levels of these lncRNAs correlate with disease progression as defined by the Response Evaluation Criteria in Solid Tumors (RECIST) [66]. The interplay of cuproptosis with other epigenetic modifications broadens our understanding of the complexity of regulatory networks in kidney cancer. Feng R. et al. developed a prognostic model to assess the risk of ccRCC based on a lncRNA signature associated with m6A modification and cuproptosis (*NFE4*, *LINC02154*, *AL161782.1*, *AL355835.1*). The *NFE4* plays a central role in this model. The study revealed that *NFE4* knockout significantly inhibits the proliferation and migratory activity of RCC cells (Caki-1 and OS-RC-2) [17].

Disulfidosis is a recently discovered form of regulated cell death that occurs during glucose deprivation due to excessive accumulation of cystine, which leads to the disruption of the cell cytoskeleton. A prognostic model was developed using 5 lncRNAs associated with disulfidosis (*FAM225B*, *ZNF503-AS1*, *SPINT1-AS1*, *WWC2-AS2*, *LINC01338*), enabling effective differentiation of patients into high- and low-risk groups for RCC. The model enables effective prediction of high or low risk of adverse patient outcomes, as well as differentiation of tumor cell mutational burden and the ability to evade the immune response in patients. It appears that suppression of *FAM225B* expression, which is actively expressed in RCC cells, reduces the migratory ability of cells (786-O, OS-RC-2) [67]. Sun et al. proposed a model consisting of 11 lncRNAs associated with disulfidosis (*AP000439.3*, *RP11-417E7.1*, *RP11-119D9.1*, *LINC01510*, *SNHG3*, *AC156455.1*, *RP11-291B21.2*, *EMX2OS*, *AC093850.2*, *HAGLR*, and *RP11-389C8.2*), according to which patients in the high-risk group are more prone to resistance to immunotherapy [68] (Figure 3).

## 5. Long Noncoding RNA in Tumor Metabolic Reprogramming

### 5.1. Glycolysis, Glutaminolysis, and the Tricarboxylic Acid Cycle

Metabolic reprogramming toward aerobic glycolysis sustains RCC survival under nutrient deprivation. lncRNAs act as direct regulators of this process, modulating the activity of key enzymes in carbohydrate and amino acid metabolism. One of the most significant mechanisms of metabolic reprogramming is the direct interaction of lncRNA with enzymes, ensuring the influx of substrates into the tricarboxylic acid cycle (TCA). Wu K. et al. found that the expression level of the *MIR4435-1HG* directly correlates with the stage of RCC and tumor size. Knockdown of *MIR4435-1HG* in 786-O and ACHN cell lines significantly inhibited cell proliferation and invasion. The use of high-tech methods (RNA pull-down combined with mass spectrometry) revealed a direct interaction between *MIR4435-1HG* and pyruvate carboxylase, a key enzyme in gluconeogenesis. This interaction ensures the replenishment of the oxaloacetate pool in the TCA cycle, supporting the energy balance and biosynthetic needs of a rapidly growing tumor [69].

Metabolic adaptation in RCC cells is characterized not only by an intensification of aerobic glycolysis but also by a profound reorganization of lipid metabolism, which is strictly regulated by specific lncRNAs. Liu et al. found that *COL18A1-AS1* acts as a tumor suppressor in ccRCC, the expression of which is suppressed due to promoter hypermethylation. The data provide evidence that *COL18A1-AS1* initiates metabolic reprogramming toward lipolysis via the *COL18A1-AS1*/miR-1286/KLF12/UCP1 axis. Consistently, the reversal of *COL18A1-AS1* silencing via 5-AZA or CRISPR/dCas9 activation led to a marked reduction in ccRCC cell viability and invasive capacity [70].

Tumors with a deficiency of TCA cycle enzymes, such as fumarate hydratase (FH) and succinate dehydrogenase, occupy a special place in the metabolic architecture of RCC. Cell survival under conditions where these pathways are inactivated becomes critically dependent on alternative carbon sources, primarily on the rate of glutaminolysis. The mechanism of adaptation to FH loss is mediated by a complex epigenetic loop centered on a specific non-coding transcript. The findings suggest that the expression of the *MIR4435-2HG* is significantly higher in RCC cells with fumarate hydratase deficiency compared to other types of RCC. *MIR4435-2HG* specifically binds to the STAT1 protein, activating the transcription of *GLS1*—a rate-limiting enzyme of glutaminolysis. Mechanistically, *MIR4435-2HG* expression is coupled to fumarate-dependent histone methylation in uok262 and pFH cells, with its inactivation resulting in suppressed glutamine metabolism. In xenograft models using immunodeficient mice, the GLS1 inhibitor CB-839 demonstrated potent anti-tumor activity, significantly suppressing the growth of FH-deficient RCC tumors [71].

An important direction of current studies is the investigation of the effect of lncRNAs on metabolic plasticity, particularly on a cell’s ability to switch to oxidative phosphorylation. Yan C. et al. found that the *MAGI2-AS3* suppresses the progression of ccRCC by modulating the *MAGI2-AS3*/miR-629-5p/PRDM16 axis. Functional experiments showed that *MAGI2-AS3* acts as a molecular sponge for miR-629-5p, preventing the post-transcriptional inhibition of the tumor suppressor PRDM16. PRDM16 is a potent regulator of metabolic plasticity, particularly the switch to oxidative phosphorylation. Notably, *MAGI2-AS3* expression is markedly downregulated in RCC tissues and continues to decline during disease progression; conversely, its restoration significantly limits cell proliferation and invasion [72].

The mechanisms by which the tumor blocks its own endogenous metabolic “brakes” to maintain a high rate of proliferation are of particular interest. A significant deficiency in *BANCR* expression was observed in RCC cells, pointing to the removal of this transcript as a key step in facilitating uncontrolled tumor growth. *BANCR* exerts a pronounced antitumor effect through direct interaction with G6PD, which is a key regulator of the pentose phosphate pathway (a branch of glycolysis). In vitro studies on RCC cells (ACHN, 786-O) demonstrated that inhibition of G6PD dimerization by *BANCR* reduces its activity and disrupts glucose metabolism, thereby blocking cell proliferation and tumor growth. These results were confirmed by in vivo experiments on a xenotransplant model, for which RCC cells (ACHN and 786-O) were grafted into immunodeficient BALB/c-nu/nu mice [1].

### 5.2. Mitochondrial Dynamics and Mitophagy

The survival of RCC cells under conditions of constant metabolic stress depends directly on the efficiency of mitochondrial quality control systems. Specific lncRNAs regulate the processes of mitophagy—the selective degradation of damaged organelles—which enables tumors to maintain a functional pool of mitochondria and avoid excessive accumulation of reactive oxygen species. An important discovery in this field was the involvement of antisense transcripts in the coordination of mitochondrial survival and autophagy signals. *LBX2-AS1* (Ladybird homeobox 2-antisense RNA 1) was overexpressed in ccRCC tissues, and its expression level was correlated with the proliferative and migratory activity of cancer cells. Mechanistically, *LBX2-AS1* acts as a regulator of the mitochondrial homeostasis transcriptional program. Knockdown of this lncRNA depletes the transcription factor FOXO3A and the simultaneous accumulation of key mitophagy marker proteins—BNIP3L and LC3. In vivo experiments confirmed that suppression of *LBX2-AS1* effectively inhibits tumor growth by preventing the adaptive degradation of damaged mitochondria, which is necessary for the progression of ccRCC [73].

### 5.3. Adaptation to Hypoxia and HIF-Dependent Mechanisms

A fundamental feature of the pathogenesis of RCC is the constitutive activation of the hypoxic response, caused by the inactivation of the tumor suppressor VHL. Under such conditions, lncRNAs form a complex regulatory network that not only enhances HIF-dependent transcription but also ensures the fine-tuning of cellular metabolic adaptation to conditions of critical oxygen deficiency.

A key feature of metabolic adaptation in RCC is the stabilization of HIF transcription factors, triggering a cascade of genes responsible for survival under hypoxic conditions. lncRNAs act here not only as targets of the hypoxic response, but also as active regulators of the stability of the HIF proteins themselves, forming stable oncogenic chains. Yang F. et al. found that knockdown of *LINC01234* in RCC cell lines (Caki-2 and A498) leads to marked inhibition of EMT. The mechanism of action of *LINC01234* is mediated through the modulation of HIF-2α transcription factor pathways, which leads to decreased expression of both HIF-2α itself and its key oncogenic targets, including VEGF-A, EGFR, c-Myc, Cyclin D1, and MET [74]. A more profound level of regulation is demonstrated by the *PVT1*, directly affecting the proteolytic degradation of hypoxia factors. Zhang et al. showed that *PVT1* promotes the migration and invasion of RCC cells (786-O, A498, 769-P), actively inducing angiogenesis. Evidence suggests that *PVT1* directly binds to the HIF2α protein, protecting it from ubiquitin-dependent degradation. Conversely, HIF2α is capable of interacting with the *PVT1* gene enhancer, thereby forming a positive feedback loop that stimulates continuous tumor growth [75].

The complexity of the hypoxic response in RCC is due to the presence of divergent regulatory pathways that control HIF-1α expression both at the translational level and via post-transcriptional silencing mechanisms. Zhao P. et al. found that *SNHG6* expression is significantly elevated in ccRCC tissues, correlating with aggressive clinical characteristics and a poor prognosis. In vitro and in vivo experiments showed that *SNHG6* acts as a potent stimulator of the metastatic potential of tumor cells, exerting its effects through direct interaction with the RNA-binding protein YBX1. This interaction leads to the selective enhancement of *HIF-1α* mRNA translation, providing tumor cells with an advantage under conditions of hypoxic stress [76]. In contrast, the *ENTPD3-AS1* exhibits pronounced tumor-suppressor properties by inhibiting hypoxic signaling. Wang et al. established that the rs67311347 polymorphism, acting as a binding site for the ZNF8 transcription factor, contributes to the activation of *ENTPD3-AS1* transcription. This lncRNA acts as a ceRNA binding to miR-155-5p and regulating the expression of HIF-1α. The data provide evidence that overexpression of *ENTPD3-AS1* suppresses proliferation in RCC cell lines (786-O and A498), while its knockdown significantly accelerates cell growth [77].

The fundamental role of hypoxic signaling in RCC pathogenesis is determined not only by the constitutive activation of HIF factors, but also by the formation of complex regulatory loops in which lncRNAs act as key mediators between oxygen sensors and embryonic development pathways. Similarly, the *HIF1A-AS2* (anti-sense RNA 2 to HIF1α) stands out as an important biomarker of ccRCC, ensuring cell survival under hypoxic conditions through direct interaction with the Gli1 protein and activation of Hedgehog signaling [78]. The clinical significance of such hypoxic networks is confirmed by the potential to develop accurate prognostic tools. Liu F. et al. proposed a model for assessing an unfavorable prognosis for the progression of ccRCC, including a number of lncRNAs (*AC011445.2*, *PTOV1-AS2*, *AP004609.3*, *SNHG19*) associated with hypoxia. *SNHG19* plays a central role in the proposed model, integrating signals from the hypoxic microenvironment with autophagy and apoptosis processes, thereby conferring a selective advantage to tumor clones during disease progression [79].

Recent studies have expanded our understanding of the oncogenic potential of lncRNA, revealing its ability to act as an epigenetic modifier and mediator between the hypoxic response and embryonic development pathways. Shao et al. detected significantly higher expression levels of *RP11-367G18.1 V2* in metastatic lesions of ccRCC, directly activated by hypoxia and HIF-1α overexpression. Mechanistically, this lncRNA interacts with the histone acetyltransferase p300, coordinating histone H4 acetylation (H4K16Ac), which critically enhances transcription of hypoxic response genes and induces EMR. In parallel, the antisense transcript *HIF1A-AS2* (antisense RNA 2 to HIF1α) emerges as an important biomarker linking hypoxia to Hedgehog signaling [80]. On the other hand, lncRNAs are capable of fine-tuning the degradation of hypoxia factors by modulating the epigenetic context of master regulators of proliferation. One example is the *FOXD2-AS1*, recruiting the MYC transcription factor to the promoter of the *EGLN3* (*PHD3*) prolyl hydroxylase gene. Although *EGLN3* normally initiates VHL-dependent degradation of HIF-1α, the functioning of the *FOXD2-AS1*/*MYC*/*EGLN3* axis in tumor conditions paradoxically accelerates proliferation and invasion, as confirmed in mouse models [81].

## 6. Long Non-Coding RNAs and the Tumor Microenvironment

### 6.1. Immune Evasion and Immune Checkpoint Regulation

The progression of RCC is inseparably linked with the creation of a local immunosuppressive environment, in which lncRNAs act as key architects of the mechanisms by which the tumor evades immune surveillance. Specific non-coding transcripts, through modulating the expression of antigen-presenting molecules and immune checkpoint ligands, enable tumor clones to effectively evade the cytotoxic action of lymphocytes and natural killer cells. Liu Q. et al. established that *LINC01232* activates tumor progression in RCC cells (786-O and Caki-1) by acting as a sponge for miR-204-5p, resulting in increased expression of *RAB22A*. RAB22A is a key regulator of intracellular trafficking, possessing pronounced pro-immunogenic functions and influencing the cell surface landscape [82]. In addition to regulating transport systems, lncRNAs constitute intricate regulatory networks coordinating the activity of classical immune evasion pathways. A regulatory panel comprising seven lncRNAs (*LINC01270*, *FIRRE*, *RP11-37B2.1*, *RP11-253I19.3*, *RP11-438L19.1*, *RP11-504P24.9*, *CTB-41I6.1*) for assessing the prognosis of overall survival in patients with advanced-stage RCC was proposed. The results indicate that increased expression of these lncRNAs positively correlates with the levels of key immune checkpoints (PD-1, PD-L1 and CTLA-4), which may indicate their role in the formation of an immunosuppressive microenvironment. This functional relationship is confirmed by the fact that knockout of the central component of the panel *LINC01270* leads to significant suppression of the proliferative and invasive activity of RCC cells (ACHN and Caki-1) [83].

Migrasomes are a class of organelles that facilitate intercellular communication. Migrasomes contain numerous signaling and bioactive molecules involved in the transfer of mRNAs and other substances between different cells via endocytosis, which has a significant impact on a wide range of physiological and pathological processes. A prognostic model was developed for lncRNAs (*ZNF503-AS1*, *NARF-IT1*, *LINC01843*, *GAS5-AS1*, *FOXD2-AS1*, *AL031985.3* and *AL162377.1*), enabling the classification ccRCC patients into high- or low-risk groups. Characteristically, the high-risk cohort exhibits more pronounced minimal residual disease and an enhanced capacity for immune evasion. Single-cell transcriptomic analysis confirmed significant cellular heterogeneity and tumor microenvironment characteristics associated with the prognostic signature [84]. Another study proposed a prognostic model comprising 12 lncRNAs (*AC009318.3*, *NOP53-AS1*, *UBE2Q1-AS1*, etc.) associated with migrasomes, demonstrating high prognostic efficacy in assessing various clinical features of metastatic ccRCC [85].

One of the most effective ways for a tumor to evade immune surveillance is through lncRNA-dependent modulation of the antigen-presenting machinery, which renders cancer cells ‘invisible’ to the effector components of the immune system. Huang et al., through analysis of the TCGA and starBase databases, identified that overexpression of the *KCNMB2-AS1* in ccRCC tissues directly correlates with advanced tumor stage, metastasis, and poor overall survival. The molecular mechanism of action is mediated via the transcription factor FOXP3, which activates the expression of the *KCNMB2-AS1* RNA, thereby sequestering miR-744-3p and preventing post-transcriptional repression of the *CD1D* target gene. Activation of the FOXP3/*KCNMB2-AS1*/*CD1D* axis not only stimulates the EMT but also forms a barrier to tumor recognition by NKT cells, as confirmed by the slowed growth of tumors in vivo following *KCNMB2-AS1* knockout [86].

### 6.2. Interaction with T Cells and Immune Infiltration

Immune infiltration in RCC creates a distinctive landscape in which a high concentration of lymphocytes is paradoxically combined with their functional inertness, allowing the tumor to progress despite the formal presence of effector cells. LncRNAs are among the participants in this process, initiating programs of T-lymphocyte functional exhaustion and transforming the anti-tumor response into a state of immunological tolerance. Tian et al. found that knocking down *SNHG1*, which is overexpressed in RCC tissues and cell lines, not only inhibits the proliferation and invasion of A498 and 786-O cells but also significantly enhances CD8+ T cells chemotaxis and cytotoxicity. *SNHG1* sequesters miR-129-3p, leading to the activation of STAT3-dependent expression of the PD-L1 ligand [87]. On the other hand, *SNHG1* maintains the overall aggressiveness of tumor clones by modulating chromatin architecture factors. Ye Z.H. et al. found that *SNHG1* acts as a ceRNA for miR-103a, regulating HMGA2 levels. The study revealed that *SNHG1* knockout or miR-103a introduction inhibits the proliferation, migration, and invasion of RCC cells, as well as enhances apoptosis, forming an *SNHG1*/miR-103a/*HMGA2* axis that contributes to disease progression [88].

In addition to the direct suppression of lymphocyte function, a critical factor in immune evasion is the restriction of cytotoxic cells’ physical access to the tumor parenchyma. Modern bioinformatic studies, combined with functional assays, enable the identification of specific transcripts responsible for the formation of such an ‘immune-repellent’ landscape. Bioinformatic analysis of the TCGA-KIRC database, combined with functional assays, revealed that *LINC00887* is overexpressed in ccRCC cells and that its expression level inversely correlates with CD8+ T-cell infiltration of the tumor. Functionally, silencing *LINC00887* was found to downregulate the immune checkpoint PD-L1 and augment CD8+ T-cell-mediated cytotoxicity, thereby positioning this RNA as a potential candidate for enhancing immunotherapeutic efficacy [89].

Overall, lncRNAs act as key architects of the RCC tumor microenvironment, establishing a complex system of immune privilege by modulating the antigen-presenting machinery CD1D and vesicular receptor transport. LncRNA coordinates the expression of immune checkpoint ligands (PD-L1, CTLA-4) and activates suppressor signaling pathways, thereby inducing functional exhaustion of T lymphocytes and limiting their chemotaxis to the tumor site. A similar lncRNA-dependent reprogramming transforms the local microenvironment into a ‘cold’ immune phenotype, which not only ensures the survival of tumor clones but also largely determines the resistance of RCC to modern immunotherapy.

## 7. The Role of Long Non-Coding RNAs in the Progression and Metastasis of RCC

### 7.1. Epithelial–Mesenchymal Transition (EMT)

EMT is a fundamental process that initiates the metastatic cascade in RCC. Upon completion of this program, tumor cells lose their apical-basal polarity and tight intercellular junctions, acquiring an invasive mesenchymal phenotype. lncRNAs act here as central regulators, coordinating the expression of epithelial state regulators and the activation of metastasis-related transcription factors.

LncRNA suppressors play a key role in inhibiting the metastatic cascade by blocking the metastatic program through the inactivation of key intracellular signaling pathways. One example is *LINC00671*, the expression level of which is significantly reduced in both tumor samples and RCC cells (769-P, Caki-1, 786-O, ACHN, and A498). The results indicate that low expression levels of *LINC00671* directly correlate with advanced stages of RCC and metastasis. Mechanistically, *LINC00671* functions as a selective ‘sponge’ for miR-221-5p, leading to the stabilization of the SOCS1 protein, a key negative regulator of the JAK/STAT pathway. Restoration of this axis effectively inhibits proliferation and the EMT in Caki-1 and 769-P cells [90]. Similar suppressive potential with regard to migratory activity is demonstrated by lncRNAs coordinating the activity of zinc-finger proteins and the state of the immune microenvironment. Regulation of the ZNF268 transcription factor is mediated by the *AC093157.1*, which, unlike the typical sequestration mechanism, promotes the maturation of miR-27a-3p (via stabilization of the DGCR8 complex) [91].

Despite the dominant role of oncogenic transcripts, the functional landscape of lncRNAs in RCC is characterized by marked heterogeneity, with individual molecules acting as tumor suppressors that inhibit the metastatic cascade. An example of such suppressor activity is the *GPRC5D-AS1*. Unlike most previously studied RNAs, it maintains the epithelial phenotype in a number of cell lines (e.g., 786-O). Functional assays demonstrated that *GPRC5D-AS1* depletion accelerates proliferation and motility in 786-O cells and leads to a marked expansion of tumor volume in vivo. At the molecular level, this phenomenon is accompanied by the induction of EMT, during which the classic ‘cadherin switch’ is observed, manifesting as increased expression of the mesenchymal marker N-cadherin, β-catenin and proliferation markers (Ki67, PCNA), alongside a simultaneous decrease in the level of the epithelial E-cadherin [2].

The miR-200/ZEB axis is a fundamental mechanism regulating the plasticity of RCC cells. It acts as an insurmountable barrier to mesenchymal transformation under normal conditions. miRNAs of the miR-200 family are considered key inhibitors of EMT, ensuring the preservation of the epithelial phenotype of cells through direct post-transcriptional repression of the transcription factors ZEB1 and ZEB2. Recent findings identified that the expression levels of a number of lncRNAs (*MALAT1*, *OIP5-AS1* and *LINC00467*) correlate with the expression of members of the miR-200 family (miR-141-3p, miR-200a-3p, miR-200b-3p, miR-200c-3p and miR-429), suggesting their involvement in a complex regulatory system of metastasis processes via the mechanism of competitive endogenous RNAs [92].

### 7.2. Invasion and Remodeling of the Extracellular Matrix

The ability of tumor cells to penetrate through the basement membrane and colonize surrounding tissues depends directly on their capacity to dynamically remodel the architecture of the extracellular matrix. lncRNAs act as key coordinators of this process, regulating both the synthesis of new matrix components and the expression of protease enzymes responsible for their degradation.

A critical stage of invasion is the ability of tumor cells to secrete enzymes that break down the physical barriers of the extracellular matrix. One of the key regulators of proteolytic activity in ccRCC cells is the *MIR155HG*. The findings suggest that the expression level of *MIR155HG* is significantly elevated in RCC cells (786-O and ACHN), and its knockdown leads to marked inhibition of migration and invasion. *MIR155HG* performs its functions by modulating both miR-155 strands (5p and 3p), which act on a wide range of genes [93]. A mechanism of invasion control via modulation of the vesicular transport apparatus was also described for *LINC01133*. Specifically, *LINC01133* was overexpressed in RCC tissues and cell lines (786-O, A498, Caki-1), where its suppression effectively impaired proliferation and migration. *LINC01133* acts via the *LINC01133*/miR-30b-5p/*Rab3D* axis, acting as a sponge for miR-30b-5p and thereby stabilizing the oncogene *Rab3D*. In view of the fact that Rab family proteins are critical for enzyme exocytosis and receptor recycling, activation of this axis provides tumor cells with the necessary dynamics to effectively progress through the remodeled matrix, as confirmed by in vivo experiments [94]. Shan et al. found that the expression of the *MEG8*, induced by the PLAG1 transcription factor, is significantly elevated in RCC tissues and cells (A498, Caki1, 786-O, 769-P and ACHN). Further investigation identified a *MEG8*/miR-495-3p/G3BP1 axis, where *MEG8*-mediated sequestration of miR-495-3p stabilizes G3BP1 to promote oncogenic progression. In vivo experiments confirmed that *MEG8* knockdown effectively inhibits tumor growth, making the PLAG1/*MEG8*/miR-495-3p/G3BP1 axis a promising target for the diagnosis and treatment of RCC [95].

The ability of tumor cells to invade is determined not only by the destruction of existing barriers but also by active modification of the extracellular matrix, creating a favorable niche for migration. One such mechanism involves the activation of specific glycosyltransferases that remodel the carbohydrate component of the matrix. *LINC01094* is proposed as one of the oncogenic RNAs mediating this mechanism in ccRCC. In vitro functional experiments showed that *LINC01094* knockdown leads to the inhibition of proliferation, migration, and invasion of RCC cells (ACHN and 769-P). In vivo experiments found that *LINC01094* suppression leads to reduced tumor growth in RCC. At the molecular level, *LINC01094* acts as a ceRNA binding to miR-224-5p and increasing the expression of the glycosyltransferase CHSY1, responsible for the synthesis of matrix components that facilitate tumor cell migration [96]. Analysis of the RNA expression profile of RCC and surrounding normal cells revealed an association of *PSMB8-AS1* with disease progression and resistance to immune checkpoint inhibitors. Significantly higher levels of *PSMB8-AS1* expression were detected in RCC tumors compared with adjacent normal tissues, correlating with a poor prognosis. *PSMB8-AS1* acts as a competing endogenous RNA, binding to miR-204-5p/miR-211 and increasing *TFAP2A* expression [97].

The functional diversity of lncRNAs enables them to act both as powerful drivers of invasion and as key suppressors of tumor growth in RCC. Fan et al. showed that lncRNA *MMP2-AS1* plays an important role in RCC progression by regulating the metastatic potential of tumor cells via the *MMP2-AS1/*miR-34c-5p/*MMP2* axis. By sequestering miR-34c-5p, *MMP2-AS1* prevents the degradation of *MMP2* mRNA, driving an aggressive oncogenic phenotype. Experimental silencing of this lncRNA effectively attenuates RCC cell (Caki-1, 786-O) invasion and migration [98]. At the same time, Yang et al. found that *APCDD1L-AS1* expression is significantly reduced in ccRCC due to hypermethylation of the lncRNA gene’s promoter region and VHL protein inactivation. Functioning as a tumor suppressor, *APCDD1L-AS1* effectively impairs RCC cell growth and metastasis by negatively regulating histone expression and inhibiting EMT [99].

The regulation of RCC progression and metastasis depends largely on the level of m6A modification of *NEAT1*. Based on the state of its methylation, *NEAT1* can act as either an oncogene or a tumor suppressor. Liu et al. found that the methyltransferase METTL14 acts as a tumor suppressor by inhibiting the activity of *NEAT1_1* via the m6A modification mechanism. It was established that METTL14 initiates the methylation of *NEAT1_1*, enabling its recognition by the protein reader YTHDF2, which triggers the degradation of this RNA. The reduction in METTL14 expression detected in RCC tissues leads to pathological stabilization and accumulation of oncogenic *NEAT1_1*, which activates the proliferation and migratory capacity of tumor cells [100]. Chen et al. found that targeted introduction of m6A marks into the *NEAT1* structure using the CRISPR/dCas13b-METTL3 system restores its expression levels in tumor cells. Functional assays revealed that induced *NEAT1* expression effectively attenuates RCC cells (786-O and OSRC) progression and migration, underscoring the critical importance of m6A-mediated stabilization for its suppressive activity [101]. These divergent findings suggest that the functional role of *NEAT1* in RCC is highly context-dependent and may be governed by the specific isoforms or methylation sites involved, with m6A modification serving as a molecular switch between oncogenic and tumor-suppressive activities.

The invasive potential of RCC cells is the dysregulation of a number of lncRNAs acting as key mediators of migration and metastasis. Meng B. et al. found that *LINC00565* maintains high levels of the metalloproteinase *ADAM19* by sequestering miR-532-3p. Available data point to knockdown of *LINC00565* in Caki-1 cells leads to a decrease in the expression level of the metalloproteinase *ADAM19*, involved in the processes of adhesion, migration, and invasion of cancer cells [102]. In addition to modulating matrix enzymes, *LINC00645* regulates intrinsic mechanisms of cell contractility by influencing the stability of key kinases. Li et al. demonstrated that *LINC00645* acts as a tumor suppressor by competing with *ROCK1* mRNA for binding to the HNRNPA2B1 protein. Under normal conditions, this competition limits the HNRNPA2B1-mediated stabilization of *ROCK1*. However, in RCC, the downregulation of *LINC00645* leads to increased *ROCK1* expression and the subsequent promotion of tumor proliferation and invasion [103]. An increased level of *MILIP* expression was detected in metastatic RCC tissues compared with primary tissues. The data indicate that silencing of *MILIP* expression reduces the migration and invasion potential of RCC cells and decreases the formation of RCC metastases in vivo. Mechanistically, *MILIP* forms an RNA-RNA duplex with the mRNA of the Snai1 family transcriptional repressor and binds to Y-box binding protein 1 YBX1, contributing to the recruitment of YBX1 and the enhancement of *Snai1* mRNA translation, playing an important role in RCC metastasis [104]. Wang et al. found that the expression of *DLEU7-AS1* is significantly elevated in RCC samples and is inversely proportional to the clinical prognosis of the disease and the expression level of miR-26a-5p. Knockdown of *DLEU7-AS1* significantly inhibits the growth and metastasis of RCC cells and slows tumor growth in vivo. Crucially, the inhibitory effects of *DLEU7-AS1* silencing were almost entirely abrogated by either miR-26a-5p inhibition or exogenous coronin-3 expression, confirming the presence of a regulatory signaling axis [105]. Pyrosequencing of *LINC00404* revealed that in RCC samples from primary tissue with distant metastases (M1) and in metastatic tissues (Mtx), significantly higher levels of *LINC00404* methylation were observed compared with RCC samples without distant metastases (M0). Notably, the GenHancer database identifies common regulatory elements between *LINC00404* and other tumor suppressor genes, including *SOX1*, *lncSOX1-5*, and *SPACA7* [106].

The clinical aggressiveness of RCC is directly determined by the activation of lncRNAs coordinating proteolytic degradation of the stroma and the dynamics of membrane trafficking, both of which are necessary for the active dissemination of tumor cells. Tokunaga T. et al. found that overexpression of the *LRRC75A AS1* is a predictor of poor prognosis in RCC, while its knockdown in RCC cells (769-P and 786-O) significantly suppresses invasion. Mechanistically, this effect is mediated via the *LRRC75A-AS1*/miR-370-5p/*ADAMTS5* axis. Sequestration of miRNA leads to the stabilization of the metalloproteinase *ADAMTS5*, facilitating the remodeling of the extracellular matrix [107]. Besides the direct degradation of extracellular matrix proteins, tumor progression is maintained through the modulation of intracellular transport systems. Cheng C. et al. found significantly higher expression levels of *Linc00239* in patient tissues and ccRCC cell lines compared to controls, correlating with an unfavorable disease prognosis. In vitro experiments on RCC cell lines (SN12C, 786-O) confirmed that *Linc00239* knockdown inhibits proliferation and metastasis. *Linc00239* functions as a molecular sponge for miR-204-5p, preventing the post-transcriptional repression of the small GTPase RAB22A. RAB22A is involved in the regulation of intracellular vesicular transport and receptor recycling [108].

Effective dissemination of tumor cells requires the constant activation of a proteolytic apparatus capable of breaking down dense stromal components. lncRNAs act here as triggers for the expression of classic ‘invasion effectors’—matrix metalloproteinases. Among such regulators, the *OSTM1-AS1* occupies a special place, as its activity directly determines the metastatic potential of RCC cells. This transcript activates proliferation and metastasis via the *OSTM1-AS1/*miR-491-5p/MMP-9 regulatory axis. The mechanism of action of *OSTM1-AS1* involves the selective sequestration of miR-491-5p, removing post-transcriptional repression of the metalloproteinase MMP-9. The unhindered degradation of the extracellular matrix induced by this cascade is a critical condition for cells to escape the primary tumor site. Experiments on cell lines (786-O, OSRC-2) and in vivo models confirmed that *OSTM1-AS1* knockdown effectively inhibits the growth and invasion of RCC tumor cells [109].

### 7.3. Tumor Stem Cells and Dedifferentiation

Carcinogenesis of RCC is largely driven by the presence of a subpopulation of tumor stem cells capable of unlimited self-renewal and exhibiting high resistance to standard therapy. LncRNAs act as key regulators of dedifferentiation, activating pluripotency transcription factors and maintaining the immature phenotype of cancer cells. The antisense RNA *NR2F2-AS1* plays a particular role in the dedifferentiation of ccRCC cells, restoring the properties of immature precursors to tumor cells. In a study of tissue samples from patients with ccRCC, a direct correlation was found between *NR2F2-AS1* levels and the expression of the small GTPase Rac1. Consistently, *NR2F2-AS1* knockdown in 786-O cells reduces Rac1 protein levels, thereby impairing the maintenance of the cancer stem cell pool. Conversely, overexpression of *NR2F2-AS1* leads to a significant increase in the tumor’s self-renewal capacity. At the molecular level, *NR2F2-AS1* is capable of suppressing the activity of miR-137, which interacts with *Rac1* mRNA; the overexpression of Rac1 enables the tumor to continuously replenish its population of aggressive, immature cells [110]. Another study showed that knockdown of *LINC02783* suppresses invasion and viability of RCC cells (786-O and ACHN), and inhibits RCC growth in vivo. The mechanism of action of *LINC02783* involves the sequestration of miR-20b, thereby activating *SOX4* expression. SOX4 is a classic transcription factor responsible for maintaining tumor stem cell properties, preserving pluripotency, and regulating dedifferentiation processes [111].

### 7.4. Vasculogenic Mimicry and Angiogenesis

The progression of RCC is inextricably linked to the formation of a well-developed vascular network, which supplies the tumor with nutrients and provides pathways for hematogenous dissemination. lncRNAs act as key determinants of neovascularization, regulating both the secretion of classical angiogenic factors and the ability of tumor cells to undergo vasculogenic mimicry. The central mechanism stimulating endothelial angiogenesis is miRNA-dependent modulation of the vascular endothelial growth factor (VEGF). Evidence demonstrates that elevated *ASMTL-AS1* levels in RCC cells correlate with VEGF induction, highlighting its essential role in driving the angiogenic switch. Ursolic acid, a natural pentacyclic triterpenoid, exhibits marked antitumor activity by specifically inhibiting *ASMTL-AS1* expression leading to a reduction in tumor angiogenic potential [112].

Besides classic angiogenesis, a key role in the aggressiveness of RCC is vasculogenic mimicry. Basically, this is the formation of functional pseudo-vascular structures by the tumor cells themselves. The *SERB* stands out as one of the drivers of this process. Experiments on the A498 and 786-O cells showed that *SERB* is recruited to the *ERβ* gene promoter, activating the ZEB1 transcription factor. This cascade endows tumor cells with high plasticity, enabling them to form pseudovessels, significantly facilitating the invasion and systemic dissemination of RCC [113].

Similar to classic angiogenesis, the activation of angiogenesis in RCC is a multi-stage process. lncRNAs act as a link between the circadian rhythms of tumor cells and the transcriptional regulation of vascular growth factors. Brooks et al. identified the circadian *ADIRF-AS1*, the expression of which is directly regulated by the BMAL1 factor and acts as a critical oncogenic driver of SCLC. Mechanistically, nuclear *ADIRF-AS1* binds to the PBAF complex, thereby neutralizing its repressor activity toward genes involved in the cell cycle and angiogenesis. Knockout of *ADIRF-AS1* in 786-O cells injected into mice leads to a complete loss of their ability to form tumor tissue [114].

Overall, lncRNAs act as master regulators of the metastatic RCC cascade, providing tumor cells with unprecedented plasticity for colonizing distant organs. lncRNAs modulate critical pathways of the EMT (miR-200/ZEB) and activate specific invasion effectors, such as metalloproteinases and cytoskeletal regulators, facilitate matrix degradation and active cell migration. Of particular significance is the role of these transcripts in maintaining the tumor stem cell pool and inducing vasculogenic mimicry, which forms an autonomous and therapy-resistant system of tumor blood supply and self-renewal.

## 8. Non-Standard Functions of lncRNA

### 8.1. Microprotein Coding

A fundamentally new level of regulation of carcinogenesis in RCC is linked to the translation of cryptic microproteins from sequences previously considered non-coding. Kun Meng et al. identified a unique oncogenic microprotein, TPM3P9, encoded by the tropomyosin 3 lncRNA pseudogene. Unlike classical lncRNAs, TPM3P9 realizes its potential not through miRNA sequestration but by directly interfering with the alternative splicing machinery. Structural and functional analyses demonstrate that the TPM3P9 microprotein associates with the RRM1 domain of RBM4, thereby preventing the excision of specific exons from the pre-mRNA of the transcription factor TCF7L2. The interaction described above leads to the accumulation of the oncogenic TCF7L2-L splice variant, which activates the NF-κB signaling pathway and induces *RELB* expression through interaction with the SAM68 protein. Further analysis confirmed that TPM3P9 overexpression in the tissues of patients with ccRCC correlates with a poor prognosis and high proliferative activity of tumor cells. The discovery of the lncRNA-microprotein axis TPM3P9/RBM4/TCF7L2-L/NF-κB axis expands our understanding of the molecular etiology of RCC, reclassifying lncRNAs from mere regulators to templates for the synthesis of biologically active proteins [115]. The functional characteristics of the SMIM26 microprotein, encoded by *LINC00493*, playing an anti-metastatic role in RCC were described. Detailed functional assays revealed that SMIM26 serves as the primary effector in suppressing RCC malignancy, where its binding to AGK and SLC25A11 impairs metabolic pathways essential for lung metastasis. SMIM26 increases the mitochondrial localization of AGK and inhibits AGK-mediated phosphorylation of AKT [116].

### 8.2. Multilevel Control Axes

The functional architecture of lncRNAs in RCC extends far beyond linear cascades, forming convergent regulatory axes capable of simultaneously influencing transcription, RNA stability, and post-translational protein modifications. Such multi-level systems enable a single lncRNA molecule to act as a central hub, coordinating the activity of epigenetic complexes, miRNA networks, and metabolic signaling pathways.

Complex multi-level regulation is demonstrated by *LINC01426*, exhibiting activity in both the cytoplasm and the nucleus of ccRCC cells. Jiang Y. et al. showed that this lncRNA not only binds to miR-423-5p, activating the *FOXM1* oncogene, but also participates in the epigenetic control of transcription. *LINC01426* was found to upregulate the transcriptional co-repressor *CTBP1* through the cytoplasmic recruitment of IGF2BP1. Further analysis confirmed that *LINC01426* directly interacts with CTBP1 in the nucleus, facilitating the recruitment of HDAC2 (histone deacetylase 2). This complex induces histone deacetylation and suppresses the transcription of miR-423-5p, forming a stable functional axis of IGF2BP1/CTBP1/HDAC2/miR-423-5p/FOXM1 that stimulates the proliferation and migration of RCC cells [117].

The epigenetic reprogramming of RCC is underpinned by the ability of specific lncRNAs acting as molecular guides to selectively direct modifying complexes to the promoter regions of target genes. A critical link in this regulation is the direct interaction of lncRNA with the catalytic subunit of the polycomb repressive complex 2 (PRC2) methyltransferase EZH2. Wang and Liu detected elevated expression of the *UFC1* in RCC tumor tissues, correlating with a poor prognosis. At the molecular level, *UFC1* interacts with EZH2, the catalytic subunit of the polycomb repressive complex 2 (PRC2), which induces trimethylation of lysine 27 of histone H3 (H3K27me3) in the promoter region of the *APC* gene, which acts as a classic tumor suppressor. Loss-of-function experiments demonstrated that depleting *UFC1* effectively suppresses cell growth and motility in ACHN and A498 cells. Chromatin immunoprecipitation (ChIP) analysis confirmed that *UFC1* suppression reduces EZH2 binding and H3K27me3 levels in the *APC* promoter, leading to the restoration of its expression [118].

Particular attention should be paid to the mechanisms by which lncRNAs act as regulators of the epigenetic status of miRNAs, forming atypical regulatory cascades. Expression profiling revealed a marked reduction in *BRE-AS1* levels in ccRCC tissues, highlighting its potential role as a tumor-suppressive transcript within these cascades. In contrast to the classical model of competitive endogenous RNAs, *BRE-AS1* suppresses the expression of miR-106b-5p by inducing methylation of the promoter region of its precursor gene, leading to the inhibition of proliferation, migration, and invasion in RCC cell lines [119].

The complexity of the multi-level pathways in RCC is also due to the formation of autonomous positive feedback loops that stabilize the oncogenic signal. Li et al. showed that *MIRE* (c-Myc-induced regulator), which is a direct transcriptional target of the *MYC* oncogene, acts as a key mediator of RCC progression. At the molecular level, *MIRE* interacts with the RNA-binding protein hnRNPK, leading to post-transcriptional stabilization of the *ELF2* transcription factor mRNA. Notably, ELF2, in turn, is capable of transcriptionally activating the expression of the *MIRE* itself. Thus, a stable ELF2–*MIRE* regulatory axis is formed, which sustains the proliferative activity of RCC cells regardless of the initial stimuli [120].

A number of studies suggest that *MAGI2-AS3* acts as a key suppressor of RCC, combining both direct protein-RNA interactions and the functions of a competitive endogenous RNA. In particular, *MAGI2-AS3* is capable of directly binding to the transcription factor HEY1, activating the transcription of the *ACY1* gene and leading to the suppression of proliferation, migration, and angiogenesis. Overexpression of *MAGI2-AS3* reduces the viability of tumor cells and inhibits the formation of vascular tubes by endothelial cells (HUVECs). The effect of suppressing tumor growth and neoangiogenesis was confirmed in vivo in xenograft experiments in BALB/c-nu/nu mice [121]. Yang et al. found that *MAGI2-AS3* also functions as a ceRNA for miR-142-3p, contributing to increased expression of the *STAM* gene. Activation of the *MAGI2-AS3*/miR-142-3p/*STAM* axis leads to the suppression of RCC cell proliferation and metastasis; conversely, overexpression of miR-142-3p or suppression of *MAGI2-AS3* promotes disease progression [122].

Other epitranscriptomic modifications that determine the stability of lncRNAs also make a significant contribution to the regulatory network of RCC. Zhou Z. et al. found that the methyltransferase WTAP acts as a key oncogenic driver in RCC, significantly accelerating the proliferation and metastasis of tumor cells. The mechanism of action of WTAP is mediated by m6A methylation of the *TEX41*, which leads to its destabilization via the recognizing reader protein YTHDF2. Under normal conditions, *TEX41* interacts with the SUZ12 protein (a component of the PRC2 complex), ensuring the epigenetic ‘silencing’ of the *HDAC1* gene. Disruption of the WTAP/*TEX41*/SUZ12/*HDAC1* regulatory axis leads to a loss of control over *HDAC1* expression, which contributes to disease progression. Notably, *TEX4*1 overexpression effectively neutralizes the pro-oncogenic effects of WTAP in 786-O and 769-P RCC cell lines [123].

lncRNAs with context-dependent functions arouse particular interest, as conflicting data regarding their role in the pathogenesis of RCC are available. Wei S. et al. identified *DRAIC* as a proto-oncogenic factor. The findings suggest that overexpression of *DRAIC* in A498 cells significantly increases proliferative potential, invasion, and migration, and also accelerates tumor growth in vivo. Within this model, *DRAIC* functions as a molecular sponge for miR-145-3p, preventing post-transcriptional repression of actin-binding protein ABRACL, responsible for cytoskeletal reorganization [124]. In contrast, Wen Y. et al. describe *DRAIC* as a tumor suppressor in clear cell RCC. *DRAIC* blocks the interaction of the hnRNPA2B1 protein with the E3 ligase FBXO11, thereby preventing proteasomal degradation. Stabilized hnRNPA2B1, in turn, promotes the degradation of m6A-modified *IGF1R* receptor mRNA, leading to the suppression of insulin-like growth factor signaling and a slowing of RCC progression [18].

An important aspect of multi-level regulation in RCC is not only the presence of lncRNAs themselves, but also the dynamic control of their stability by specialized protein complexes that determine the accessibility of epigenetic modifiers to chromatin. Yang et al. identified a specific role for the Nuclear Exosome Targeting Complex (NEXT) in controlling the accessibility of the polycomb complex PRC2. Under normal conditions, the repressive PRC2 complex ensures the epigenetic silencing of genes via trimethylation of lysine 27 of histone H3 (H3K27me3). The RNA-binding protein ZCCHC8, a key component of the NEXT complex, serves as a pivotal driver of tumor progression in ACHN models. The mechanism involves the targeted degradation of specific G4/U-rich lncRNAs, which under physiological conditions block PRC2 binding to chromatin, acting as a ‘shield’ for promoters. Pathological activation of ZCCHC8 leads to excessive degradation of these lncRNAs and the release of the catalytic subunit EZH2, inducing abnormal methylation and epigenetic silencing of key tumor suppressors, such as *SEMA5A* and *ARID1A*. Clinical evidence suggests that tumors with high ZCCHC8 expression demonstrate increased sensitivity to the EZH2 inhibitor tazemetostat, positioning ZCCHC8 as a promising biomarker for personalized targeted therapy in ccRCC [125].

Thus, the presented data demonstrate that lncRNAs in RCC perform significantly more complex and diverse functions than previously assumed, going beyond the scope of classical regulatory models. In this context, lncRNAs are capable not only of mediating gene expression regulation but also of encoding functional microproteins that directly intervene in key cellular processes, such as splicing and signaling pathways. lncRNAs form multi-layered regulatory axes. These axes integrate epigenetic, transcriptional, and post-transcriptional mechanisms. Further complexity arises from the dynamic regulation of the stability of the lncRNAs themselves and their context-dependent functionality, which may result in opposing effects under different conditions.

## 9. The Role of Long Non-Coding RNAs in Patient Sensitivity to RCC Treatment

In recent years, a conceptual understanding has emerged that drug resistance in RCC is not the result of a single dominant mechanism, but rather the complex interplay of transcriptional, post-transcriptional, and metabolic regulatory networks. RCC is characterized by high heterogeneity and a tendency to develop resistance to both targeted therapy and chemotherapy and radiotherapy. Against this backdrop, lncRNAs are regarded as key regulators of tumor biology, capable of modulating gene expression, signaling pathways, and cellular processes, including apoptosis, autophagy, and metabolism.

### 9.1. Sunitinib

The largest number of studies is devoted to investigating the role of lncRNA in the sensitivity of RCC patients to treatment with the tyrosine kinase inhibitor sunitinib. Liu et al. found that *SNHG12* is actively expressed in RCC tissues and cells insensitive to sunitinib. In vivo experiments showed that active expression of *SNHG12* promotes tumor growth, while suppression of *SNHG12* expression restores the sensitivity of tumor cells to sunitinib. The mechanism of *SNHG12* action involves interaction with the SP1 protein, preventing its ubiquitin-dependent degradation and leading to increased *CDCA3* expression [126]. Comparative analysis revealed a substantial increase in *MALAT1* expression within sunitinib-resistant RCC cells. Functional inhibition of *MALAT1* was found to overcome this resistance by acting on the miR-362-3p/G3BP1 signaling pathway [127]. Shi et al. revealed that the nuclear receptor TR4 regulates the expression of the *TASR*, stabilizing *AXL* mRNA and thereby contributing to the development of resistance to sunitinib. Further analysis confirmed that blocking TR4 in RCC cells (OSRC-2 and SW839) using tretinoin, metformin, or TR4-shRNAs enhances the sensitivity of the cells under investigation to sunitinib and slows tumor progression, a finding confirmed in a preclinical mouse model [128]. Analysis of lncRNA expression in tumor and non-malignant tissue samples from patients with metastatic RCC revealed that high expression of *TUSC7* is associated with low sensitivity to sunitinib and poorer survival [129] (Table 2).

Saimaiti et al. established that exosomes derived from sunitinib-resistant RCC cells (R-exos) promote cell proliferation and increase the expression of proliferation-related genes (*CCND1*, *PCNA*), as well as suppressing apoptosis and the expression of Bax and Caspase-3 proteins in sunitinib-resistant RCC cells (RCC/R) via the delivery of *SNHG16*. In a xenograft model (CDX-R), R-exosomes were found to stimulate tumor growth in vivo, whereas the knockdown of exosomal *SNHG16* effectively reduces RCC oncogenesis. *SNHG16* promotes cell proliferation and inhibits apoptosis via regulation of the miR-106a-5p/TROAP axis [130]. Xiao et al. found that the *MATN1-AS1*, localized predominantly in the cytoplasm, promotes metastasis of clear cell RCC and resistance to sunitinib via the miR-214-5p/E2F2 axis. Silencing *MATN1-AS1* reduces proliferation, migration, invasion, and EMT, and enhances sensitivity to sunitinib [131].

Chen et al. found that the TCL6148 peptide, encoded by the *TCL6*, exerts an antitumor effect by inducing ferroptosis in RCC cells via the GOT1/GPX4 signaling pathway and significantly enhancing the therapeutic efficacy of sunitinib. At the molecular level, the action of the TCL6148 peptide promotes the accumulation of Fe^2+^, increases levels of reactive oxygen species and enhances lipid peroxidation, making RCC cells more susceptible to ferroptosis [132]. In another earlier study, the authors found significantly lower levels of *TCL6* expression in RCC tissues compared with controls, correlating with lower overall and recurrence-free survival rates in patients. Experiments in RCC cell lines (786-O, Caki-1) demonstrated that upregulated *TCL6* increases paclitaxel sensitivity via miR-221 suppression. Conversely, *TCL6* silencing reduces drug efficacy, highlighting its potential in combinatorial therapy [133].

Autophagy regulation is one of the key mechanisms underlying the development of drug resistance. Li et al. showed that *HOTAIR* acts as a ceRNA by suppressing miR-17-5p, leading to increased expression of the key autophagy regulator *Beclin1*. Stimulation of autophagic processes leads to increased survival in RCC cells and reduced sensitivity to sunitinib therapy [134]. Of particular interest is the *IGFL2-AS1*, involved not only in intracellular regulation but also in the intercellular transmission of sunitinib resistance in RCC. Competitively binding to the multifunctional RNA-binding protein hnRNPC, *IGFL2-AS1* leads to increased expression of *TP53INP2*, resulting in the activation of autophagy and the development of resistance to sunitinib. At the same time, the delivery of *IGFL2-AS1* antisense oligonucleotides via chitosan nanoparticles successfully overcomes sunitinib resistance in patient-derived RCC xenografts [135]. Tian et al. found that the lncRNA *SNHG1* also acts as an oncogenic factor, promoting tumor progression and resistance to sunitinib through the activation of autophagy. The data indicate that increased expression of *SNHG1* in RCC cells is associated with a poor prognosis for patients, whilst knockdown of this lncRNA suppresses tumor growth and eliminates resistance to sunitinib and autophagy in RCC cells. This effect is mediated by the interaction of the *SNHG1* with PTBP1 and the subsequent activation of ATG7, a key protein in the autophagy process [136].

### 9.2. Sorafenib

Sorafenib is a targeted anticancer drug acting as a multi-kinase inhibitor, suppressing angiogenesis and tumor proliferation by inhibiting the binding of serine/threonine kinases to receptor tyrosine kinases. Luciferase reporter and functional assays showed that *PLK1S1* suppresses the expression of miR-653, targeting the *CXCR5* chemokine receptor. However, inhibition of miR-653 negates the anti-tumor effect of *PLK1S1* knockdown, thereby restoring RCC progression and cell resistance to sorafenib. A study of BALB/c-nu nude mice implanted with human renal adenocarcinoma (ACHN) cells demonstrated that *PLK1S1* knockdown significantly slows tumor growth compared with the control group [137]. Jin et al. demonstrated that increased expression of *KIF9-AS1* in RCC cells (Caki-1 and 786-O) leads to increased cell viability, an increase in the sorafenib IC50 value, and reduced apoptosis. The target of *KIF9-AS1* is miR-497-5p; the TGF-β signaling pathway and autophagy are proposed as downstream effectors, serving as key factors in chemoresistance in RCC [138]. Wang et al. found that the expression level of the *EMS* (E2F1 messenger RNA stabilizing factor) is significantly higher in sorafenib-resistant RCC tissues and cell lines than in sorafenib-sensitive RCC tissues and cell lines, and that knockdown of *EMS* increases the sensitivity of sorafenib-resistant renal cell carcinoma cells to this drug. In vivo studies showed that suppression of *EMS* expression in combination with sorafenib treatment significantly slows the progression of RCC. Evidence suggests that *EMS* sequesters miR-363-3p, which negatively regulates the expression of dual-specificity phosphatase 10 *(DUSP10*) by acting on its 3’-untranslated region. The authors note that the *EMS*/miR-363-3p/*DUSP10* axis demonstrates a direct link between lncRNA and the regulation of drug response and may serve as a prognostic and therapeutic marker [139].

### 9.3. Axitinib

Axitinib is a member of the group of targeted anticancer drugs, acting as a highly selective inhibitor of VEGF receptor tyrosine kinases. Pan et al. showed that *STX17-DT* contributes to reduced sensitivity to axitinib by decreasing the accumulation of mitochondrial ROS and suppressing ferroptosis. *STX17-DT* stabilizes *IFI6* mRNA through interaction with hnRNPA1. IFI6 (Interferon Alpha Inducible Protein 6) is a protein encoded by one of the interferon-stimulated genes (ISGs) that promotes tumor cell survival. Molecular analysis confirmed that *STX17-DT* can be transmitted via extracellular vesicles, spreading drug resistance to neighboring cells. The findings suggest that the use of siRNA against STX17-DT in combination with axitinib *in vivo* in NCG mice with a model of RCC significantly enhances the therapeutic effect of the drug, indicating the potential of this approach to overcome resistance [140].

### 9.4. NVP-BEZ235

One of the new targeted anticancer drugs is NVP-BEZ235, which is a synthetic low-molecular-weight compound effectively inhibiting the catalytic activity of class 1 PI3K and mTOR, and demonstrating significant anticancer activity against various types of cancer cells, including RCC. Chen et al. showed that *CHKB-AS1* was associated with resistance to NVP-BEZ235 treatment in RCC. In vivo experiments in nude mice with an RCC model confirmed that overexpression of *CHKB-AS1* significantly enhances tumor growth and increases its resistance to NVP-BEZ235, whilst knockdown of *CHKB-AS1* leads to a marked reduction in tumor volume and weight. The mechanism of action of the *CHKB-AS1* is linked to direct interaction with the MAP4 protein, a key activator of the PI3K/AKT/mTOR signaling pathway, which leads to reduced treatment efficacy and enhanced tumor growth [141].

### 9.5. Chemo- and Radiotherapy

The efficacy of chemotherapy and radiotherapy in RCC is largely determined by the state of intracellular regulatory networks capable of blocking drug-induced apoptosis. Zhao et al. demonstrated that *LINC00667* promotes ccRCC progression and cisplatin resistance through a series of in vitro and in vivo experiments. Mechanistically, *LINC00667* sequesters miR-143-3p, thereby upregulating the EMT driver ZEB1, while the ectopic restoration of miR-143-3p effectively attenuates these oncogenic effects [142]. In addition to chemoresistance and targeted therapy resistance, lncRNAs also play a significant role in the development of tumor cell radioresistance. One of the key regulatory hubs in this process is the transcription factor YY1, which is involved in the control of DNA repair. *LINC02532* plays an important role in the regulation of YY1-dependent mechanisms in RCC. *LINC02532* acts as a competing endogenous RNA that sponges miR-654-5p to regulate *YY1* expression. Functionally, *LINC02532* expression promotes radioresistance, whereas its depletion sensitizes RCC cells to radiotherapy both in vitro and in xenograft models [143].

### 9.6. Other Medications

Ropivacaine is used in oncology as an amide-type local anesthetic for epidural analgesia and peripheral nerve blocks, providing effective pain relief during the perioperative period and in palliative care. Xiong et al. found that the elevated expression of *RMRP* in RCC cells (786-O, Caki-1) is blocked by the administration of the local anesthetic ropivacaine; however, *RMRP* knockdown enhances the inhibitory effect of ropivacaine on RCC cells. Further analysis confirmed that *RMRP* directly interacts with the histone methyltransferase EZH2, regulating the methylation of the histone *CCDC65* and suppressing its expression. These findings indicate that ropivacaine exerts its anti-tumor effects—inhibiting RCC growth, migration, and invasion—specifically via the *RMRP*/EZH2/*CCDC65* axis [144]. Shen et al. found that lower levels of *LINC02154* expression in tumor tissues from patients with ccRCC correlated with higher sensitivity to pazopanib, sorafenib, sunitinib, and temsirolimus. Consistently, *LINC02154* knockdown was found to inhibit ACHN cell proliferation and migration by suppressing the expression of *FDX1* and *DLST* genes closely associated with cuproptosis and the tricarboxylic acid cycle [145].

In recent years, publicly available data and bioinformatics approaches have been increasingly used to develop models capable of predicting the response of patients with RCC to treatment. Li et al., in their analysis of TCGA data, identified a profile of lncRNAs (*LUCAT1*, *LINC01138*, *LINC01605,* and *HOTAIR*) associated with glycolysis that reliably predicts survival in patients with clear cell RCC. The authors note that the high-risk group exhibits a higher level of immune cell infiltration and greater efficacy of immunotherapy compared to the low-risk group [146]. Hu et al. developed a prognostic model based on five lncRNAs (*AC002070.1*, *AC092953.2*, *AC103706.1*, *LINC01943,* and *LINC02027*) associated with mitophagy, which stratifies patients into groups based on sensitivity to specific drugs using data from TCGA. Mitochondriophagy is a cellular process involving the removal of damaged mitochondria, which helps maintain energy homeostasis and prevents the accumulation of oxidative stress. Notably, patients in the low-risk category demonstrate enhanced anti-tumor immune activity and high sensitivity to bortezomib, whereas the high-risk group exhibits immune suppression, an elevated tumor mutation burden, and increased sensitivity to EGFR and TGF-β inhibitors [147]. It was demonstrated that high expression of the IL-2 receptor gamma subunit (*IL2RG*) correlates with poor survival and the activation of immune pathways in clear cell RCC. A signature of lncRNAs associated with IL2RG (*LINC00944*, *AC016773.2*, *LINC02446*, *LINC02328*, *U62317.2*, *KIF1C-AS1*), which identifies patients with high sensitivity to sunitinib and temsirolimus [148].

lncRNAs continue to play a central role in research into the mechanisms of RCC treatment, encompassing both the classic and most common types of RCC, as well as rare subtypes, such as RCC with *NONO-TFE3* gene rearrangement, characterized by a high degree of malignancy and a poor prognosis. Chen et al. found that the *NMRK2* enhances mitochondrial respiration in RCC cells with *NONO-TFE3* gene rearrangement, acting as a molecular ‘scaffold’ to sustain tumor metabolism. Combined *NMRK2* suppression and metformin treatment in UOK109 cells proved more effective than individual interventions, further establishing *NMRK2* as a potential target for combinatorial treatment [149].

Overall, an analysis of recent studies indicates that lncRNAs are key regulators of drug sensitivity in RCC, exerting their effects through a wide range of mechanisms, including ceRNA interactions, regulation of autophagy, ferroptosis, epigenetic modifications, and metabolic re-programming. A key point is that many lncRNAs are simultaneously involved in multiple signaling pathways, forming complex regulatory networks. From a practical standpoint, this justifies the need to develop combined therapeutic strategies aimed at simultaneously targeting multiple regulatory nodes. However, to confirm the data described above, extensive clinical validation of the identified targets is required.

## 10. Conclusions

lncRNAs represent a fundamental regulatory layer in RCC, governing oncogenic transformation across all levels of gene expression. They regulate RCC pathogenesis at all levels, from epigenetic chromatin modification and transcriptional control to post-transcriptional processes, including alternative splicing, RNA stability, and translation. Their ability to function as molecular scaffolds, guides, miRNA sponges, and regulators of protein complexes underlies their central role in the formation of complex regulatory networks within tumor cells.

Despite progress in understanding lncRNAs in RCC, unresolved fundamental and applied challenges still hinder their clinical translation. One important aspect is the quantitative and systematic interpretation of ceRNA networks. Most existing studies focus on linear axes (lncRNA–miRNA–mRNA), whereas in reality, regulation is network-based with multiple feedback loops. There is a need for the integration of multidimensional data (transcriptomics, epigenomics, proteomics), the construction of dynamic models of regulatory networks, and the use of systems biology and machine learning methods to identify key nodes associated with disease pathogenesis. The issue of reproducibility of results requires particular attention. A significant proportion of the data has been obtained from limited samples of cell lines or in vivo models, which do not always reflect the heterogeneity of RCC in patients. The lack of standardization in methodologies (expression normalization, selection of control groups, experimental design) makes it difficult to compare the results of different studies. A significant limitation is the lack of functional validation of the identified lncRNAs. Many studies demonstrate a correlation between expression and clinical parameters; however, causal relationships remain unclear. An important unresolved issue remains the study of lncRNA interactions with epigenetic and post-transcriptional mechanisms, including m6A modification, alternative splicing, and the formation of transcriptional condensates. To date, there is no comprehensive understanding of how these levels of regulation integrate with one another to form a unified regulatory landscape of the tumor cell.

From a clinical perspective, the prospective direction for lncRNA usage is as a biomarker for disease development and progression, as well as for assessing patient response to therapy. However, there are a number of limitations here, such as the low specificity of individual molecules, variability in expression between patients, and the influence of pre-analytical factors (type of biomaterial, storage conditions). The development of multi-gene signatures and their validation in large, independent patient cohorts is required. Of particular interest is the therapeutic targeting of lncRNAs using antisense oligonucleotides, small interfering RNAs (siRNAs), and CRISPR technologies. Nevertheless, realizing this potential is complicated by the need to address critical challenges in ensuring effective and selective delivery to tumor cells, minimizing off-target effects, and achieving a sustained therapeutic response, which is further complicated by the high structural and functional variability of the non-coding transcripts themselves.

The main objective of this review was to analyze publications from the past few years focusing on the role of lncRNAs in RCC pathogenesis, allowing us to examine the latest concepts and most recent data on the subject. The focus on the current scientific landscape comes with certain limitations that must be taken into account when conducting a comprehensive assessment of the data. Firstly, prioritizing recent publications ensures maximum relevance but may result in insufficient coverage of classic fundamental works that laid the foundations of functional genomics. Secondly, the heterogeneity of methodological approaches, including differences in study design, sequencing platforms, sample selection criteria, and data normalization methods, makes it difficult to directly compare results and carry out meta-analytical integration. However, it is precisely this focus on current research that allows us to form a modern understanding of the rapidly evolving landscape of the non-coding genome. The systematization of the data obtained not only reveals new regulatory mechanisms underlying RCC pathogenesis but also opens up real prospects for the development of innovative methods for early diagnosis and the creation of personalized targeted therapy strategies that meet the challenges of modern medicine.

Overall, lncRNAs represent a key component of the molecular architecture of RCC, integrating various levels of gene expression regulation and signaling pathways. Further study of lncRNAs is predicted to advance our understanding of the mechanisms of carcinogenesis and lay the foundation for the development of new personalized diagnostic and therapeutic strategies aimed at improving treatment outcomes for patients with RCC.

## Figures and Tables

**Table 1:** The regulatory role of long non-coding RNAs in modulating key signaling pathways in RCC.

Name of lncRNA	Role	Basic Mechanism/Regulatory Axis	Biological Effect	References
*LINC00944*	Oncogene	Modulation of Akt phosphorylation	Regulation of RCC cells’ proliferation and migration	[23]
*LINC00460*	Oncogene	Activation of the signaling pathway PI3K/AKT/mTOR	Stimulation of proliferation, migration, and invasion of RCC cells, inhibition of apoptosis, induction of epithelial–mesenchymal transition (EMT)	[24]
*RCAT1*	Oncogene	*RCAT1/*miR-214-5p/*E2F2* → PI3K/AKT/mTOR	Stimulation of RCC cells’ proliferation, migration, and invasion, inhibition of apoptosis	[25]
*FTX*	Oncogene	*FTX*/miR-4429/*UBE2C* → PI3K/AKT/mTOR	Activation of CDK1 and CDK6, growth of tumor mass and volume	[26]
*AGAP2-AS1*	Oncogene	m6A-stabilization (IGF2BP3)/miR-9-5p/*THBS2* → PI3K/Akt	Stimulation of M2 polarization in macrophages, tumor growth	[15]
*FGD5-AS1*	Oncogene	miR-5590-3p → ERK/AKT → mTORC1	Metabolic reprogramming, metastasis	[27]
*TCL6*	Suppressor	miR-155/Src/Akt → EMT	Inhibition of RCC cell migration, induction of apoptosis	[28]
*PVT1*	Oncogene	miR-328-3p/*FAM193B* → MAPK/ERK and PI3K/AKT	Stimulation of RCC cell proliferation, tumor growth	[29]
*GABPB1-IT1*	Suppressor	miR-21/*PTEN* (antagonist PI3K/AKT/mTOR)	Inhibition of RCC cell proliferation, migration, and invasion, induction of apoptosis, suspension of cell cycle at the G0/G1 phase	[30]
*MSC-AS1*	Oncogene	miR-3924/*WNT5A* → Wnt/β-catenin	Stimulates RCC cell proliferation and migration	[32]
*NLGN1-AS1*	Oncogene	miR-136-5p/*FZD4* → Wnt/β-catenin	Accumulation of β-catenin, acceleration of tumor growth	[33]
*SLERCC*	Suppressor	UPF1 → Wnt/β-catenin	Inhibits proliferation, metastasis, and angiogenesis in RCC cells	[34]
*LINC00160*	Oncogene	Inhibition of *IL20RB* expression → activation of Wnt and mTOR	Activates Wnt, mTOR, and fatty acid metabolism, promotes tumor growth and chemoresistance	[35]
*LINC01939*	Suppressor	miR-154 sequestration → inactivation of the Wnt/β-catenin and Notch pathways	Inhibition of proliferation and migration, induction of apoptosis in RCC cells	[36]
*SNHG12*	Oncogene	miR-30a-3p/*RUNX2*, *WNT2*, *IGF-1R*	Stimulates the proliferation and migration of RCC cells; high levels of *SNHG12* expression correlate with a poor clinical prognosis	[37]
*LINC00675*	Suppressor	Inhibition of the Wnt/β-catenin pathway (reduction in β-catenin levels)	Decreased proliferative, invasive, and migratory capacity of RCC cells	[38]
*MIR503HG*	Suppressor	Epigenetic and m6A-mediated suppression of the *NOTCH1*/*VEGFC* axis involving H2A.Z and HNRNPC	Inhibits lymphangiogenesis, RCC cells migration, and lymphatic metastasis	[40]
*RP11-567G11.1*	Oncogene	Activation of Notch signaling by modulation of the Jagged1/HES5/HEY1 axis	Stimulates the proliferation and invasion of RCC cells, inhibits apoptosis, and reduces sensitivity to cisplatin	[41]
*ARSR*	Oncogene	Exosomal axis *ARSR*/miR-34/miR-449/*STAT3*	Induces polarization of macrophages in the M2 phenotype, stimulates cytokine secretion, angiogenesis, and the proliferation of RCC cells	[42]
*MIAT*	Oncogene	The core assembly of the *MIAT* scaffold with the ETS1 factor to induce the JAK3/STAT3 pathway	Stimulates the exhaustion of CD8+ T cells and enhances the proliferation, migration, and invasion of RCC cells	[43]
*LINC01605*	Oncogene	IGF2BP2-dependent stabilization of *JAK3* mRNA → activation of STAT3 signaling and induction of membrane sialylation (ST6GALNAC5↑)	Enhances cell membrane sialylation, induces exhaustion of CD8+ T cells, and increases the proliferation and invasion of RCC cells	[44]
*LINC01671*	Suppressor	Regulation of purine metabolism and modulation of the MAPK, NF-κB, mTOR, PI3K-Akt, and Wnt pathways	Inhibits the proliferation and migration of RCC cells and induces apoptosis	[45]
*LUCAT1*	Oncogene	miR-375/YAP1 (Hippo pathway effector)	Stimulates the proliferation and migration of RCC cells	[46]
*LINC02609*	Oncogene	Activation of the MAPK signaling pathway (by increasing ERK phosphorylation)	Stimulates the proliferation, migration, and invasion of RCC cells	[47]

**Table 2:** The effect of lncRNA on treatment response in RCC.

Name of lncRNA	Drug/Type of Treatment	Role in Therapy	Mechanism/Regulatory Axis	Biological Effect	References
*SNHG12*	Sunitinib	Resistance	*SNHG12/*SP1/*CDCA3*	Increased survival and aggressiveness of RCC cells, inhibition of apoptosis in sunitinib treatment	[126]
*MALAT1*	Sunitinib	Resistance	*MALAT1*/miR-362-3p/G3BP1	Increased viability and invasiveness of RCC cells, suppression of apoptosis, and enhanced chemoresistance to sunitinib	[127]
*TASR*	Sunitinib	Resistance	TR4/*TASR*/*AXL*	Development of resistance to sunitinib and maintenance of RCC cell proliferation and migration during treatment	[128]
*TUSC7*	Sunitinib	Resistance	High *TUSC7* levels correlate with poor response; the mechanism depends of p53 pathway status	Promotion of tumor cell survival and proliferation during treatment with targeted therapies, significant reduction in time to progression and overall survival	[129]
*SNHG16*	Sunitinib	Resistance	*SNHG16/*miR-106a-5p/TROAP	Providing survival and proliferation of tumor cells despite the effects of sunitinib, blocking drug-induced apoptosis, and accelerating disease progression during treatment	[130]
*MATN1-AS1*	Sunitinib	Resistance	*MATN1-AS1*/miR-214-5p/E2F2	Development of resistance to sunitinib, promotion of metastasis through activation of the EMT, and increased proliferation and invasion of RCC cell	[131]
*TCL6*	Sunitinib	Sensitivity	The micropeptide TCL6148 induces ferroptosis via the GOT1/GPX4 pathway	Enhanced therapeutic effectiveness of sunitinib; promotion of Fe2+ and ROS accumulation, and lipid peroxidation in RCC cells	[132]
*TCL6*	Paclitaxel (Chemotherapy)	Sensitivity	*TCL6*/miR-221	Enhanced drug-induced apoptosis, a significant reduction in viability, and inhibition of RCC cell proliferation during treatment	[133]
*HOTAIR*	Sunitinib	Resistance	*HOTAIR/*miR-17-5p/*Beclin1*	Increased survival of RCC cells due to activation of protective autophagy, development of resistance to sunitinib, and stimulation of tumor growth	[134]
*IGFL2-AS1*	Sunitinib	Resistance	*IGFL2-AS1*/hnRNPC/TP53INP2 → autophagy	Activation of protective autophagy, providing cell survival during therapy; the transfer of resistance to sensitive cells via extracellular vesicles	[135]
*SNHG1*	Sunitinib	Resistance	*SNHG1*/PTBP1/*ATG7* → activation of autophagy	Development of resistance to sunitinib, stimulation of proliferation, migration, and invasion of RCC cells, and activation of protective autophagy, enabling tumor survival under therapeutic stress	[136]
*PLK1S1*	Sorafenib	Resistance	*PLK1S1*/miR-653/*CXCR5*	Induction of resistance to sorafenib, stimulation of proliferation, and invasion of RCC cells	[137]
*KIF9-AS1*	Sorafenib	Resistance	*KIF9-AS1*/miR-497-5p/TGF-β, autophagy	Increased survival of RCC cells and Sorafenib IC50 values, suppression of therapy-induced apoptosis	[138]
*EMS*	Sorafenib	Resistance	*EMS/*miR-363-3p/*DUSP10*	Increased survival and proliferation of RCC cells during treatment; inhibition of apoptosis; accelerated tumor growth in vivo under therapeutic conditions	[139]
*STX17-DT*	Axitinib	Resistance	*STX17-DT*/hnRNPA1/*IFI6*/ferroptosis	Inhibition of drug-induced ferroptosis, stimulation of tumor growth, and weight	[140]
*CHKB-AS1*	NVP-BEZ235	Resistance	*CHKB-AS1*/MAP4/PI3K/AKT/mTOR	Induction of resistance to the NVP-BEZ235 drug, increased RCC cells proliferation, and stimulation of tumor volume and weight growth in vivo	[141]
*LINC00667*	Cisplatin (DDP)/Chemotherapy	Resistance	*LINC00667*/miR-143-3p/*ZEB1*	Reduced sensitivity to cisplatin, stimulation of RCC cell proliferation, migration, and invasion, inhibition of apoptosis, and activation of EMT	[142]
*LINC02532*	Radiation therapy	Resistance	YY1/*LINC02532*/miR-654-5p	Promotion of double-strand DNA break repair, inhibition of apoptosis, and maintenance of cell viability following radiation exposure	[143]
*RMRP*	Ropivacaine	Oncogene	Ropivacaine/*RMRP*/EZH2/CCDC65, Ropivacaine inhibits *RMRP*, activating the tumor suppressor *CCDC65*	Stimulation of proliferation, migration, and invasion of RCC cells; inhibition of apoptosis	[144]
*LINC02154*	Targeted therapy (pazopanib, sorafenib, sunitinib, temsirolimus)	Sensitivity predictor (biomarker) and oncogene driver	*LINC02154*/*FDX1*/*DLST*	Stimulation of RCC cell proliferation and migration; suppression of cuproptosis by inhibiting key enzymes *FDX1* and *DLST*	[145]

## Data Availability

No new data were created or analyzed in this study. Data sharing is not applicable to this article.

## Supplementary Materials

The following supporting information can be downloaded at: https://www.mdpi.com/article/10.3390/ijms27115071/s1.

## Abbreviations

The following abbreviations are used in this manuscript:

RCC	Renal cell carcinoma
ccRCC	Clear cell renal cell carcinoma
pRCC	Papillary renal cell carcinoma
RNA	Ribonucleic acid
mRNA	Messenger RNA
miRNA	MicroRNA
lncRNA	Long non-coding RNA
pri-miRNA	Primary microRNA
ceRNA	Competing endogenous RNA
MRE	miRNA response element
UTR	Untranslated region
RISC	RNA-induced silencing complex
TAD	Topologically associated domain
EMT	Epithelial–mesenchymal transition
m6A	N6-methyladenosine
RNP	Ribonucleoprotein
TCA	Tricarboxylic acid cycle
ROS	Reactive oxygen species
GSH	Glutathione
HIF	Hypoxia-inducible factor
VHL	Von Hippel–Lindau tumor suppressor
TCGA	The Cancer Genome Atlas
GSEA	Gene Set Enrichment Analysis
PDX	Patient-derived xenograft
NEXT	Nuclear Exosome Targeting Complex
RECIST	Response Evaluation Criteria in Solid Tumors
