# Supplementary material for: Long Non-Coding RNAs in Pathogenesis of Renal Cell Carcinoma: Epigenetic Regulation, Signaling Pathways, and Therapeutic Strategies
Source: Int J Mol Sci. 2026 Jun 3;27(11):5071. doi: 10.3390/ijms27115071 (PMC13256571; doi:10.3390/ijms27115071)
Supplement: Supplementary file 1 [file ijms-27-05071-s001.zip › ijms-4318742-supplementary.pdf]

Table S1. Functional landscape and mechanisms of action of lncRNA in RCC

| Name of lncRNA | Role | Main Mechanism / Regulator Axis | Key biological effect | References |
|---|---|---|---|---|
| *NORAD* | Oncogene | miR-144-3p / *MYCN* | Stimulation of RCC cells proliferation and migration | [48] |
| *EMBP1* | Oncogene | miR-9-5p / CCNE2 / E2F1 | Stimulates the proliferation, migration and invasion of RCC cells, activates the EMT, and enhances the stem cell potential of cells by inducing the expression of the KLF4 and Nanog | [49] |
| *TTN-AS1* | Oncogene | miR-195 / Cyclin D1 | Stimulates proliferation and accelerates the G1/S transition in the cell cycle of RCC cells | [50] |
| *LINC00894* | Oncogene | miR-660-5p / *TPX2* | Stimulates the proliferation, migration and invasion of pRCC cells | [51] |
| *LINC00478* | Oncogene | Transcriptional activation of *PBX3* and induction of CDCA8/CDK2 cyclins | Stimulates the proliferation, migration and invasion of RCC cells, accelerates the cell cycle and promotes tumor growth | [52] |
| *PCGEM1* | Oncogene | miR-433-3p / *FGF2* | Stimulates proliferation and migration, and inhibits apoptosis in RCC cells | [53] |
| *LINC00641* | Oncogene | miR-340-5p / *c-Myc*, CyclinD1, MMP-2 | Stimulates proliferation, colony formation and invasion, and inhibits apoptosis in RCC cells | [54] |
| *SNHG4* | Oncogene | miR-204-5p / *RUNX2* | Stimulates the proliferation and invasion of RCC cells, inhibits apoptosis by reducing the activity of caspases-3, -8 and -9 and increasing Bcl-2 levels | [55] |
| *SNHG3* | Oncogene | miR-10b-5p / *BIRC5* | Stimulates the proliferation, migration and invasion of RCC cells | [56] |
| *SNHG3* | Oncogene | *SNHG3* / miR-139-5p / *TOP2A* → modulation of CTLA-4 and recruitment of Tregs / M0 macrophages | Stimulates the proliferation and migration of RCC cells | [57] |

| | | | | |
|---|---|---|---|---|
| *SNHG12* | Oncogene | miR-200c-5p / *COL11A1* | Stimulates the viability and invasion of RCC cells and inhibits apoptosis | [58] |
| *SNHG12* | Oncogene | KMT2B / H3K4me3 / *SNHG12* / *E2F1* / *CEP55* | Induction of neoangiogenesis and aggressive metastatic growth | [59] |
| *SNHG16* | Oncogene | miR-1301-3p / *STARD9* | Stimulates the proliferation of RCC cells and inhibits apoptosis | [60] |
| *SNHG17* | Oncogene | miR-328-3p / *H2AX* | Stimulates the proliferation, migration and invasion of RCC cells, inhibits apoptosis and promotes tumor growth | [61] |
| *DARS-AS1* | Oncogene | miR-194-5p / *DARS* | Stimulates the proliferation and suppresses the apoptosis of RCC cells | [62] |
| *CYTOR* | Oncogene | miR-136-5p / *MAT2B* / *BAG3* | Stimulates the proliferation and invasion and inhibits apoptosis of RCC cells | [63] |
| *ZFAS1* | Suppressor | miR-185-5p / *SLC25A28* | Induction of ferroptosis | [64] |
| *MIR4435-1HG* | Oncogene | Direct interaction with pyruvate carboxylase | Stimulates the proliferation, migration and invasion of RCC cells, positively regulates the expression of pyruvate carboxylase, influencing tumor metabolism | [69] |
| *COL18A1-AS1* | Suppressor | miR-1286 / *KLF12* / *UCP1* | Inhibits the proliferation and metastasis of RCC by stimulating lipid breakdown (causing tumor cells to 'shrink') | [70] |
| *MIR4435-2HG* | Oncogene | Fumarate / H3K4me3 / *MIR4435-2HG* / *STAT1* / *GLS1* | Metabolic remodeling: activation of glutamine metabolism and glycolysis, stimulation of RCC cell proliferation and migration | [71] |
| *MAGI2-AS3* | Suppressor | miR-629-5p / *PRDM16* | Inhibits the proliferation, migration and aggressiveness of RCC cells | [72] |
| *BANCR* | Suppressor | Direct interaction with G6PD → inhibition of G6PD dimerisation → suppression of pentose phosphate pathway activity | Inhibits the proliferation of RCC cells and induces apoptosis, significantly inhibiting tumor growth in vivo by modulating glucose metabolism | [1] |

| | | | | |
|---|---|---|---|---|
| *LBX2-AS1* | Oncogene | *FOXO3A* / *BNIP3L* / LC3 | Stimulates the proliferation and migration of RCC cells by inhibiting mitophagy, contributing to tumor survival | [73] |
| *LINC01234* | Oncogene | Modulation of HIF-2$\alpha$ pathways (regulation of HIF-2$\alpha$, VEGFA, EGFR, c-Myc, Cyclin D1 and MET levels) | Stimulates proliferation, migration, invasion and EMT in RCC cells | [74] |
| *PVT1* | Oncogene | *PVT1* stabilizes HIF2$\alpha$ by preventing its degradation, while HIF2$\alpha$ activates *PVT1* transcription | Stimulation of the proliferation, migration, invasion and metastasis of RCC cells, induction of tumor angiogenesis | [75] |
| *SNHG6* | Oncogene | Interaction with YBX1 → enhanced translation of HIF1$\alpha$ | Stimulates the proliferation and metastasis of RCC cells | [76] |
| *ENTPD3-AS1* | Suppressor | SNP rs67311347 (G>A) → binding ZNF8 → *ENTPD3-AS1* / miR-155-5p / HIF-1$\alpha$ | Inhibits the proliferation of RCC cells | [77] |
| *RP11-367G18.1 V2* | Oncogene | Hypoxia/HIF-1$\alpha$ / interaction with p300 / increased H4K16Ac on the *Twist1*, *SLUG* and *VEGF* promoters | Stimulates the EMT, proliferation and metastasis of RCC cells | [80] |
| *FOXD2-AS1* | Oncogene | Recruits the MYC transcription factor to the *EGLN3* gene promoter | Stimulates the proliferation, migration and invasion of RCC cells | [81] |
| *LINC01232* | Oncogene | miR-204-5p / *RAB22A* | Stimulates the proliferation, migration and invasion of RCC cells | [82] |
| *KCNMB2-AS1* | Oncogene | FOXP3 / miR-744-3p / *CD1D* | Stimulates proliferation, migration, invasion and EMT of RCC cells | [86] |
| *SNHG1* | Oncogene | miR-129-3p / STAT3 / PD-L1 | Stimulates the proliferation and invasion of RCC cells, and inhibits the cytotoxicity and cytokine secretion (IFN-$\gamma$, TNF-$\alpha$, IL-2) of CD8+ T cells | [87] |
| *SNHG1* | Oncogene | *SNHG1* / miR-103a / *HMGA2* | Stimulates the proliferation and invasive capacity of RCC cells, inhibits apoptosis | [88] |

| | | | | |
|---|---|---|---|---|
| *LINC00887* | Oncogene | Suppression of chemokine secretion (CXCL9, CXCL10, CXCR3) and activation of immunosuppressive molecules (PD-L1, PD-1, TGFB1, IDO1 | Blocks the infiltration of CD8+ T cells into the tumour, reduces their cytotoxicity and chemotaxis, contributing to the progression of RCC | [89] |
| *LINC00671* | Suppressor | miR-221-5p / *SOCS1* | Inhibits the proliferation, migration and invasion of RCC cells, blocks the EMP | [90] |
| *AC093157.1* | Oncogene | *AC093157.1* stabilizes DGCR8 to facilitate miR-27a-3p maturation, leading to the downregulation of the tumor suppressor *ZNF268* | Inhibits apoptosis and reduces tumor infiltration by T-helper cells and memory T cells, whilst simultaneously recruiting suppressor Treg cells | [91] |
| *GPRC5D-AS1* | Suppressor | Suppression of EMT and inhibition of the Wnt/β-catenin pathway | Inhibition the proliferation, migration and invasion of RCC cells; reduction in tumor volume and mass in vivo | [2] |
| *MIR155HG* | Oncogene | *MIR155HG* acts as a precursor to miR-155-5p / miR-155-3p | Stimulates the proliferation, migration and invasion of RCC cells, and increases the levels of proliferation markers (KI67, PCNA) and metastasis markers (MMP-2, MMP-9) | [93] |
| *LINC01133* | Oncogene | miR-30b-5p / Rab3D | Enhances the proliferation, migration and invasion of RCC cells and promotes tumor growth in vivo | [94] |
| *MEG8* | Oncogene | PLAG1 / miR-495-3p / G3BP1 | Stimulates the proliferation, migration and invasion of RCC cells and promotes tumor growth in vivo | [95] |
| *LINC01094* | Oncogene | FOXM1 / *LINC01094* / miR-224-5p / *CHSY1* | Stimulates proliferation, migration, and EMT induction (increased levels of N-cadherin, Snail, and MMP2) in RCC cells, promotes tumor growth and metastasis in vivo | [96] |
| *PSMB8-AS1* | Oncogene | miR-204-5p (miR-211) / *TFAP2A* | Stimulates the proliferation and invasion of RCC cells | [97] |
| *MMP2-AS1* | Oncogene | miR-34c-5p / *MMP2* | Stimulates the proliferation, migration, and invasion of RCC cells, promotes tumor growth in vivo | [98] |

| | | | | |
|---|---|---|---|---|
| *APCDD1L-AS1* | Suppressor | Epigenetic regulation of histones and modulation of the VHL/HIF1$\alpha$ axis | Antimetastatic effect—inhibition of EMF and metastasis to the lungs, induction of apoptosis (Caspase-3 / Bcl-2) | [99] |
| *NEAT1_1* | Oncogene | METTL14-mediated m6A methylation normally causes *NEAT1* degradation. In tumors, low methylation → *NEAT1* accumulates → cancer progresses. | Regulation (stimulation in the absence of METTL14) of RCC cell proliferation and migration | [100] |
| *NEAT1* | Suppressor | m6A methylation (METTL3) increases *NEAT1* expression | Reduced proliferation and migratory capacity of RCC cells | [101] |
| *LINC00565* | Oncogene | miR-532-3p / ADAM19 | Stimulates the proliferation, migration, and invasion of RCC cells, and activates the EMF | [102] |
| *LINC00645* | Suppressor | *LINC00645* competitively binds to the HNRNPA2B1 protein, reducing the stability of *ROCK1* mRNA | Inhibits the proliferation, migration, and invasion of RCC cells | [103] |
| *MILIP* | Oncogene | TFAP2C / *MILIP* / YBX1 / Snai1 | EMF stimulation, enhanced migration and invasion of RCC cells, development of distant metastases | [104] |
| *DLEU7-AS1* | Oncogene | miR-26a-5p / coronin-3 | Stimulation of proliferation, enhancement of migration and invasion of RCC cells, and inhibition of apoptosis | [105] |
| *LRRC75A-AS1* | Oncogene | miR-370-5p / *ADAMTS5* | Stimulation of the proliferation and invasion of RCC cells, promoting tumor progression and metastasis | [107] |
| *Linc00239* | Oncogene | miR-204-5p / *RAB22A* | Stimulation of the proliferation, migration, and invasion of RCC cells | [108] |
| *OSTM1-AS1* | Oncogene | miR-491-5p / *MMP-9* | Stimulation of the proliferation, migration, and invasion of RCC cells | [109] |
| *NR2F2-AS1* | Oncogene | *NR2F2-AS1* / Rac1 | Increased stemness of RCC cells | [110] |

| | | | | |
|---|---|---|---|---|
| *LINC02783* | Oncogene | miR-20b / Sox-4 | Increased viability of RCC cells, stimulation of invasion, and promotion of overall tumor progression | [111] |
| *ASMTL-AS1* | Oncogene | Ursolic acid / *ASMTL-AS1* / HuR / VEGF | Stimulation of proliferation, migration, invasion, and angiogenesis of RCC cells. Ursolic acid inhibits tumor growth by reducing the expression of ASMTL-AS1 | [112] |
| *SERB* | Oncogene | *SERB* / ERβ / ZEB1 | Stimulation of vasculogenic mimicry formation and enhancement of RCC cells cell invasion | [113] |
| *ADIRF-AS1* | Circadian oncogenic RNA | *ADIRF-AS1* regulated by circadian factors and directly interacts with components of the chromatin-remodeling complex PBAF, modulating the expression of extracellular matrix genes | Regulation of the circadian rhythm of oncogenes, stimulation of tumorigenesis in vivo | [114] |
| *TPM3P9* | Oncogene | The microprotein TPM3P9 binds to the splicing factor RBM4 blocking alternative splicing of exon 13 in the *TCF7L2* gene | Stimulation of cell proliferation and RCC growth, activation of NF-κB inflammatory signaling | [115] |
| SMIM26 | Suppressor | The microprotein SMIM26 interacts with the kinase AGK and the regulator SLC25A11, retaining AGK in mitochondria and inactivating the AKT pathway | Inhibition of tumor growth and metastasis to the lungs, and maintenance of mitochondrial respiration and glutathione transport | [116] |
| *LINC01426* | Oncogene | In the cytoplasm, *LINC01426* recruits IGF2BP1 to stabilize CTBP1 mRNA; in the nucleus, *LINC01426* serves as a scaffold for the CTBP1 protein, enhancing the transcriptional repression of miR-423-5p, which leads to the activation of FOXM1 | Stimulation of RCC cell proliferation and migration, promotes tumor growth in vivo | [117] |

| | | | | |
|---|---|---|---|---|
| *UFC1* | Oncogene | *UFC1* interacts with EZH2 protein, enhancing H3K27me3 enrichment in the promoter region of *APC* gene, leading to its epigenetic repression | Enhanced proliferation and migratory capacity of RCC cells | [118] |
| *BRE-AS1* | Suppressor | *BRE-AS1* reduces the expression of miR-106b-5p by enhancing the methylation of its precursor gene | Inhibition of RCC cell proliferation and migration | [119] |
| *MIRE* | Oncogene | *c-Myc* / *MIRE* / hnRNPK / ELF2,<br>ELF2–MIRE | The development and progression of RCC | [120] |
| *MAGI2-AS3* | Suppressor | *MAGI2-AS3* / HEY1 / ACY1 | Suppression of RCC cell viability and migration, inhibition of tumor angiogenesis | [121] |
| *MAGI2-AS3* | Suppressor | *MAGI2-AS3* / miR-142-3p / STAM | Inhibition of the proliferation and metastasis of RCC cells | [122] |
| *TEX41* | Suppressor | The methyltransferase WTAP induces m6A modification of *TEX41*, leading to its degradation via YTHDF2; reduced *TEX41* levels attenuate SUZ12 activity, thereby releasing the repressive effect on the HDAC1 | Inhibition of the proliferation and metastasis of RCC cells | [123] |
| *DRAIC* | Oncogene | *DRAIC* / miR-145-3p / ABRACL | Enhanced proliferation, migration, and invasion of RCC cells, promote tumor growth in vivo | [124] |
| *DRAIC* | Suppressor | *DRAIC* / hnRNPA2B1 / FBXO11 axis → IGF1R mRNA degradation | *DRAIC* prevents FBXO11-mediated degradation of hnRNPA2B1. Stabilized hnRNPA2B1 promotes the degradation of m6A-modified IGF1R mRNA, suppressing IGF signaling and slowing RCC progression | [18] |
